# Multiple Dataset-Based Insights into the Phylogeny and Phylogeography of the Genus *Exbucklandia* (Hamamelidaceae): Additional Evidence on the Evolutionary History of Tropical Plants

**DOI:** 10.3390/plants14071061

**Published:** 2025-03-29

**Authors:** Cuiying Huang, Qiang Fan, Kewang Xu, Shi Shi, Kaikai Meng, Heying Du, Jiehao Jin, Wei Guo, Hongwei Li, Sufang Chen, Wenbo Liao

**Affiliations:** 1 State Key Laboratory of Biocontrol and Guangdong Provincial Key Laboratory of Plant Resources, School of Life Sciences, Sun Yat-sen University, Guangzhou 510275, China; hcying7@mail.sysu.edu.cn (C.H.); fanqiang@mail.sysu.edu.cn (Q.F.); doing1998@foxmail.com (H.D.); jinjh3@mail2.sysu.edu.cn (J.J.); 2 Co-Innovation Center for Sustainable Forestry in Southern China, Key Laboratory of National Forestry and Grassland Administration on Subtropical Forest Biodiversity Conservation, College of Life Science, Nanjing Forestry University, Nanjing 210037, China; xukw10@njfu.edu.cn; 3 College of Forestry and Landscape Architecture, South China Agricultural University, Guangzhou 510642, China; shis@scau.edu.cn; 4 Guangxi Key Laboratory of Quality and Safety Control for Subtropical Fruits, Guangxi Subtropical Crops Research Institute, Nanning 530001, China; mengkk@mail2.sysu.edu.cn; 5 College of Horticulture and Landscape Architecture, Zhongkai University of Agriculture and Engineering, Guangzhou 510250, China; gwei@zhku.edu.cn; 6 Guangdong Geological Survey Institute, Guangzhou 510080, China; lihongw1981@126.com

**Keywords:** *Exbucklandia*, Hamamelidaceae, phylogeny, phylogeography

## Abstract

Southeast Asia’s biodiversity refugia, shaped by Neogene–Quaternary climatic shifts and the Tibetan Plateau uplift, preserve relict lineages like *Exbucklandia* (Hamamelidaceae). Once widespread across ancient continents, this genus now survives in Asian montane forests, offering insights into angiosperm diversification. Chloroplast haplotypes formed three clades—Clade I (*E. tricuspis*), Clade II (*E. populnea*), and Clade III (*E. tonkinensis*)—with *E. longipetala* haplotypes nested within II/III. Nuclear microsatellites (SSRs) identified two ancestral gene pools: *E. populnea* and *E. tricuspis* showed predominant ancestry in Pool A, while *E. tonkinensis* and *E. longipetala* were primarily assigned to Pool B. All taxa exhibited localized genetic admixture, particularly in sympatric zones. Divergence dating traced the genus’ origin to tropical Asia, with northward colonization of subtropical China ~7 Ma yielding *E. populnea* and *E. tonkinensis*. Quaternary Glacial Cycles triggered southward expansions, chloroplast capture, and localized hybridization. Morphological, nuclear, and plastid molecular evidence supports reclassifying *E. longipetala* as *E. populnea* × *E. tonkinensis* hybrids lacking genetic cohesion and *E. tricuspis* as a distinct species with a mixed nuclear composition. This study highlights how paleoclimate-driven gene flow shaped the phylogeography of relict taxa in Southeast Asia and the urgency of habitat restoration to conserve *Exbucklandia*.

## 1. Introduction

Neogene palaeogeographical changes and Quaternary climatic fluctuations have significantly influenced the genetic structure and divergent lineages of modern Southeast Asian plants [1,2,3,4]. During the Quaternary period, many plant species persisted in very limited locations or faced severe constraints, as population expansion and migration were strongly hindered by montane orogenesis and recurring glacial periods [5,6,7]. These enduring plant taxa, which survived extreme geological and climatic events, are referred to as “relict” taxa or “living fossils” (such as *Ginkgo biloba* Linnaeus and *Cathaya argyrophylla* Chun & Kuang). They are believed to be the sole surviving members of once-diverse taxa and provide valuable insights into species extinction and diversification, with their distribution patterns becoming ‘hotspots’ for biogeography and conservation biology [8,9,10,11,12,13]. The uplift of the Tibetan Plateau has facilitated the formation of the Asian monsoon climate, significantly impacting the climate of South China and influencing local plant distribution patterns [14,15,16,17,18,19]. The establishment of the East Asian monsoon system led to a transition from arid to humid conditions across most parts of South China, allowing paleotropical forests to expand from southern coastal areas into northern inland regions (i.e., from Malaysia–Indonesia region to Southeast China) [20,21,22,23]. Consequently, the plant communities in East and South China possess intricate historical origins and harbor a vast array of diverse plant species. This rich diversity makes the region an excellent subject for studying plant species diversity and historical population dynamics in the context of global climatic and geological changes.

*Exbucklandia* R. W. Brown (Hamamelidaceae) is a kind of relict trees currently found only in tropical–subtropical evergreen broadleaved forests (EBLFs) of East and South Asia, primarily at altitudes of 1000 m or higher [24,25,26,27]. Fossil records indicate that *Exbucklandia* was once widely distributed across the warm temperate zones of both the New World and the Old World, with its origins dating back to no later than the Tertiary period, specifically since the Late Cretaceous [28,29,30,31,32,33,34,35,36,37]. Additionally, Exbucklandioideae represents an early diverging lineage within Hamamelidaceae, consisting solely of the genus *Exbucklandia*. As an ancient and genetically unique monophyletic lineage, *Exbucklandia* holds significant importance for paleobotanists and modern botanists in exploring the origins and early evolution of angiosperms [24,38,39,40,41,42].

**Figure 1:**
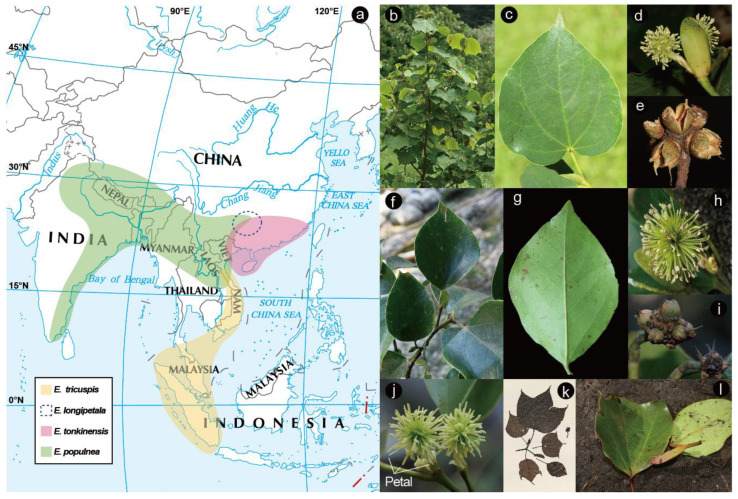
Distributions and morphological diversity of genus *Exbucklandia*. (**a**) Map of distributions based on the global specimens’ records; (**b**–**e**) *E. populnea*; (**f**–**i**) *E. tonkinensis*; (**j**) *E. longipetala*; (**k**,**l**) *E. tricuspis*.

*Exbucklandia* comprises four recognized species according to *Flora Reipublicae Popularis Sinicae* [43] (Figure 1): *Exbucklandia tonkinensis* (Lecomte) H. T. Chang is distributed sporadically in the mountainous evergreen forests of southern and southwestern China, as well as northern Vietnam. It is representative of the typical flora found in the southern subtropical evergreen broad-leaved forests. *Exbucklandia populnea* (R. Br. ex Griff.) R. W. Brown has a wide distribution in the montane evergreen forests of Southwest China, as well as in India, Myanmar, and Thailand. *Exbucklandia longipetala* H. T. Chang, which is documented in the southern Guizhou and northern Guangxi regions of China, contributes to the diversity of the genus within its limited range. *Exbucklandia tricuspis* (Hall.) Chang was published by H. T. Chang in Acta Scientiarum Naturalium Universitatis Sunyatseni (1959, 1973) as a new combination of several specimens collected in the Malaysia–Indonesia region [44,45]. Even though *E. tricuspis* has not been listed in certain taxonomic databases, such as Tropicos [46] and the World Flora Online (WFO) Plant List [47], it is a validly published species under the International Code of Nomenclature (ICN) [48].

Taxonomic studies on *Exbucklandia* have primarily concentrated on morphological characters, leading to some controversy among botanists regarding the classification of certain species. For instance, in H. T. Chang’s study, *E. tricuspis* (Figure 1k,l) is distinguished by its small trilobed leaves, narrow stipules (up to 4 cm × 8 mm), and capitulate infructescences bearing 8–13 capsule features, which distinctly differentiate it from *E. populnea,* found in continental Asia. However, it has been taxonomically merged into *E. populnea* in other botanical works [38,49]. Additionally, *E. longipetala* was first described as a new species in 1959 due to its distinctly elongated petals [43,44,45] (Figure 1j), but this characteristic has also been observed in wild populations of both *E. populnea* and *E. tonkinensis*. In the study of fossil taxa, comparative analyses of morphology and distribution between *E. acutifolia* J. Huang et Z. K. Zhou sp. nov., a fossil foliage reported in Yunnan province, China, and the modern *Exbucklandia* species suggest that the distribution boundaries of *Exbucklandia* have shifted over time [37,42]. Specifically, there appears to be a trend of Sino-Himalayan expansion and Sino-Japanese recession in the distribution of these species.

Currently, there is a lack of genomic data for *Exbucklandia* species, and constructing a ddRAD-seq library has proven challenging due to the rapid degradation of its total DNA material. In this study, we sampled a total of 59 populations representing all four species in the genus *Exbucklandia* across southeastern Asia. Then, we conducted PCR amplification of 21 SSR markers across 50 populations (14–27 individuals of each) and of 4 cpDNA regions across 56 populations (8 individuals of each) to investigate their population genetics. In addition, we applied shallow-genome sequencing technology to one representative sample from each population to obtain plastid genomes. Using these data, we constructed plastid phylogenetic relationships and population genetic structure of nuclear genes (SSRs). Through these efforts, we aimed to achieve two main objectives: (1) clarifying the taxonomic boundaries of the four *Exbucklandia* species by integrating morphological and molecular data; (2) investigating how historical geological and climatic factors have influenced the current distribution patterns of *Exbucklandia* species.

## 2. Results

### 2.1. Haplotype Network of the Combined Chloroplast Regions

**Figure 2:**
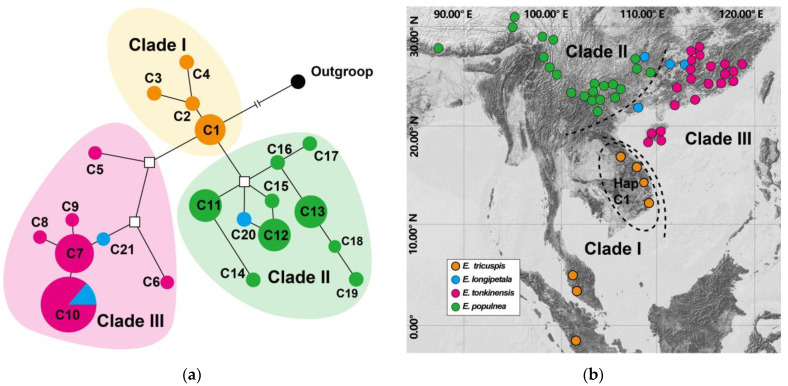
Results of haplotype network analysis based on four chloroplast fragments of 56 populations. (**a**) Network of 21 haplotypes of *Exbucklandia* cpDNA fragments. Circle size represents the number of populations that share specific chloroplast haplotypes; different colors represent the proportions of different species. The numbers assigned to the circles are arbitrary haplotype numbers. White squares in the network indicate missing intermediate haplotypes that were not found in the samples analyzed. (**b**) The geographical distribution of the 56 populations, along with the distributions of the 21 cpDNA haplotypes. Different colors of dots represent different species.

Four chloroplast fragments (*trnS*–*psbZ, trnG*–*trnfM*–*rps14, trnV, and rpl32*) were successfully amplified in 56 of 59 populations (eight individuals of each population) of all four *Exbucklandia* species. After alignment and trimming, the combined-sequence length was 2345 bp without gaps. The combined dataset, incorporating outgroup sequences (*Rhodoleia championii* Hook. f.), was trimmed to 2401 bp and employed for haplotype network reconstruction. Each population was detected to have only one chloroplast haplotype, and a total of twenty-one haplotypes were detected, with 25 single-nucleotide variants (SNVs) (Tables S1 and S2). Haplotype network analysis (Figure 2a) showed that four haplotypes were detected in *E. tricuspis* forming Clade I, nine unique haplotypes in *E. populnea* forming Clade II, and six haplotypes in *E. tonkinensis* forming Clade III. For *E. longipetal*, three haplotypes were observed across the four populations sampled: one haplotype was grouped in Clade II and two in Clade III. The geographical distribution of these haplotypes (Figure 2b) indicated distinct patterns: Clade I was located in Southern Asia, Clade II was present in Southwest China, and Clade III was found in Southeast China. Notably, haplotype C1, which was positioned centrally in the haplotype network, was recorded in the four populations sampled from Vietnam.

### 2.2. Population Genetics Based on SSR Loci

Of the 24 SSR loci analyzed, 21 loci showed consistent amplification across more than 90% of populations, while 3 loci were excluded from further analysis due to PCR amplification efficiencies below 50%. For these 21 SSR loci, a total of 244 alleles were detected across the 50 populations (14–27 individuals of each), with 6–18 alleles at each locus (Table S3). These alleles showed similar allele size ranges compared to previous research on the development of these SSR markers [50], supporting the reliability of these markers used in this study. Among these 50 populations, Shannon’s Diversity Index (I) ranged from 0.16 to 1.22 (mean 0.649 ± 0.242); the observed heterozygosity (*H_O_*) ranged from 0.072 to 0.481 (mean 0.296 ± 0.107), the expected heterozygosity (*H_E_*) ranged from 0.198 to 0.547 (mean 0.372 ± 0.092), and the fixation index (*F*) ranged from −0.158 to 0.422 (mean 0.152 ± 0.141) (Table 1).

*The F*-statistics of the 21 SSR loci, performed to analyze the genetic diversity of the whole genus and of the different groups of samples, are revealed in Table 2 (the statistical results of each locus are shown in Table S4). Genetic differentiation (*F_ST_*) ranged from 0.432 (±0.023) in *E. tonkinensis* to 0.557 (±0.04) in *E. populnea*, with moderate-to-high divergence (mean *F_ST_* = 0.507–0.536). Inbreeding coefficients (*F_IS_*) were highest in *E. tricuspis* (0.283 ± 0.07) and lowest in *E. longipetala* (0.116 ± 0.063). Gene flow (*N_M_*) was notably higher in *E. tonkinensis* (0.367 ± 0.037) compared to other species. Notably, the overall fixation index (*F_IT_*) consistently exceeded *F_ST_* and *F_IS_* values in all taxa, highlighting substantial genetic structure both within and among populations.

**Figure 3:**
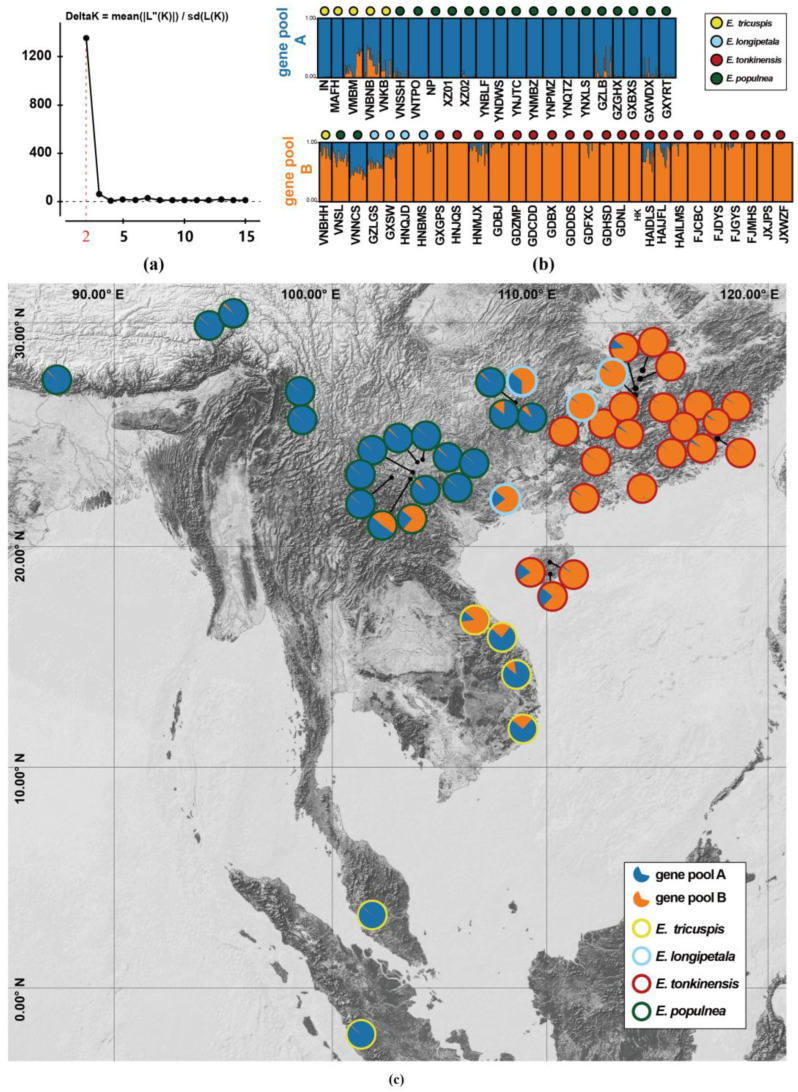
Genetic structure analysis using STRUCTURE HARVESTER. (**a**) ∆K values obtained using STRUCTURE HARVESTER based on combinations of 21 SSR markers. ∆K = 2 was the most likely value, suggesting the presence of two gene pools for all 4 *Exbucklandia* species (50 populations). (**b**) Classification of 50 populations into two gene pools, where the *x*-axis shows accessions and the *y*-axis shows the probability (from 0 to 1). The membership of the accessions is indicated by different colors (pool A, blue; pool B, orange). Species that accessions belong to are labeled above accessions with dots of different colors, and population names are labeled under accessions. (**c**) Geographical distribution of two gene pools’ frequencies in different populations. The colors in the pie chart represent different gene pool components, and the border colors of pies represent different species.

The structure analysis conducted on the 50 populations of *Exbucklandia* revealed that the optimal ΔK value was two (Figure 3a). The populations were divided into two distinct genetic pools (Figure 3b,c): *E. populnea* was derived mostly from gene pool A, while *E. tonkinensis* harbored mostly gene pool B. In areas bordering the distribution of these species, such as in Northern Vietnam and Southern China, populations exhibited an admixture of these two gene pools. For the four populations of *E. longipetala*, the two located near *E. populnea* (GZLGS and GXSW) showed an admixture of the two gene pools, while the other two populations near *E. tonkinensis* in Hunan province were derived mostly from pool B, as were most of the *E. tonkinensis* populations. For *E. tricuspis*, the two populations in Malaysia–Indonesia region were predominantly assigned to pool A components; all four populations in Vietnam exhibited an admixture of pools A and B, with three dominated by pool A and one showing a higher proportion derived from pool B.

### 2.3. Phylogenetic Trees and Divergence Time Estimation

Finally, the total DNA extracts from 34 representative individuals (out of the 59 individuals total) met the requirements for high-throughput sequencing. Approximately 6 Gb of raw data was obtained from each of the 34 *Exbucklandia* individuals, and plastid genomes were successfully assembled for all of them (GenBank accession numbers: PV132455–PV132488, Table S5). These plastid genomes exhibited a typical quadripartite structure, with lengths ranging from 160,554 to 160,905 bp (Table S5). No significant structural differences were observed among individuals from different taxa or geographical distributions.

**Figure 4:**
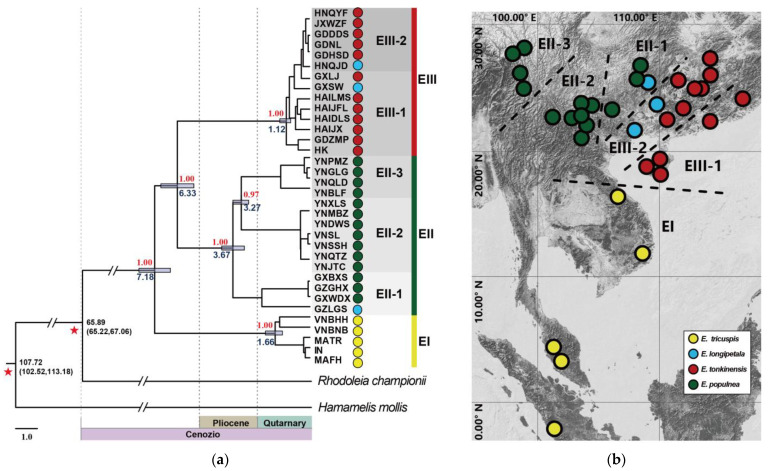
(**a**) BEAST-derived chronograms of the 34 *Exbucklandia* repetitive individuals based on cpDNA genomes with two calibration points (red pentastar). Mean divergence dates for major nodes are labeled with blue bars indicating 95% HPD clade credibility intervals for nodes of particular interest with ages under the bar (in MA). Bayesian posterior probabilities are sequentially labeled above nodes. (**b**) Geographic distribution of 34 repetitive individuals and clades we divided by BEAST-derived chronograms. Colors of dots represent different species.

Phylogenetic reconstruction and divergence time estimation were conducted using *Rhodoleia championii* Hook. f. and *Hamamelis mollis* Oliv. as outgroups, revealing that the most recent common ancestor (TMRCA) of extant *Exbucklandia* species diverged approximately 7.18 million years ago (MA) (Figure 4a). A Bayesian inference (BI) tree constructed with 34 *Exbucklandia* individuals and multiple outgroups confirmed that *Exbucklandia* and *Rhodoleia* together form a basal lineage of Hamamelidaceae (Figure S1). Subsequent BI and maximum likelihood (ML) analyses of these *Exbucklandia* individuals, with *R. championii* as the outgroup, demonstrated topological congruence with the time-calibrated phylogeny (Figures S2 and S3). The time of the most recent common ancestor (TMRCA) of all the extant *Exbucklandia* was estimated to be 7.18 MA. The samples of *E. tricuspis*, *E. tonkinensis*, and *E. populnea* formed three distinct monophyletic groups, designated as clades EI, EII, and EIII, with their respective TMRCA estimated at 1.66 MA, 3.67 MA, and 1.12 MA. Clades EII (*E. populnea*) and EIII (*E. tonkinensis*) exhibited a sister relationship, diverging at ~6.33 MA, and subsequently clustered with clade EI (*E. tricuspis)*. The three samples of *E. longipetala* did not form a monophyletic group: one clustered within clade EII, and two within clade EIII. Clade EII can be further divided into three subclades (EII-1, EII-2, and EII-3), which were sampled from the eastern, central, and western distribution areas of *E. populnea* (Figure 4b). Clade EIII was further divided into two subclades: EIII-1 and EIII-2. The six samples in subclade EIII-1 were sampled from Hainan and coastal areas of Guangdong, while samples in subclade EIII-2 were collected from inland areas of Guangdong and neighboring provinces (Figure 4b).

## 3. Discussion

### 3.1. The Origin and Dispersal History of Exbucklandia by Seed Dispersal

Our phylogenetic dating using plastid genomes estimates the divergence of the *Exbucklandia*-*Rhodoleia* clade from other Hamamelidaceae lineages at 107.72 MA (95% HPD: 102.5234, 113.1766 MA), consistent with prior divergence estimates (about 85–110 MA) [42,51,52]. However, the TMRCA of *Exbucklandia* and *Rhodoleia* exhibits notable discrepancies across studies: the result of this study was estimated at 65.89 MA (95% HPD: 65.22–67.06 MA), in contrast to earlier estimates of about 80 MA [51], 55 MA [42], and 37 MA [52]. These variations likely arise from differential molecular dataset selection (nuclear vs. plastid gene evolutionary rate heterogeneity), molecular clock model (strict vs. relaxed), or taxon sampling strategies affecting node calibration. Crucially, the Late Cretaceous (>65 MA) fossil records of both *Exbucklandia* and *Rhodoleia* strongly conflict with the younger estimates of 55 MA and 37 MA. Regarding extant *Exbucklandia* species, the TMRCA was inferred to be 7 Ma—substantially younger than previous estimates of about 14–15 MA [42,51]. This disparity primarily reflects sampling scale differences that earlier studies employed limited *Exbucklandia* representatives (1–2 samples) alongside cross-genus and family data for joint calibration, whereas our analysis incorporated 34 complete plastid genomes for genus-specific temporal reconstruction. The narrower confidence intervals (55–70 MA vs. broader ranges in prior studies) and median estimate position further support the robustness of our dating framework.

Fossil records of lamina, flowers, and fruits of *Exbucklandia* have been extensively documented in East Asia and North America, dating from the Oligocene to the Pliocene [28,29,30,31,32,33,34,35,36,37], which indicates that there was once a broader global distribution of this genus during the Cenozoic. Currently, only four extant species of *Exbucklandia* are restricted to East Asia, with their TMRCA estimated to be 7.18 MA. This evidence supports the hypothesis that global cooling during the Late Tertiary contributed to the extinction of all *Exbucklandia* species in North America and most species in East Asia [25,35,37].

Mature capsules of *Exbucklandia* exhibit rapid curling and eject seeds ballistically over short distances during dehiscence under mechanical pressure. This dispersal mechanism with limited seed dispersal distance provides a slow seed dispersal (compared to pollen dispersal) in many tree populations, as observed in *Juglans* [53], *Populus* [54], and *Quercus* [55]. Consequently, the plastid lineage mediated directly by seed dispersal could provide valuable insights into the origin and dispersal of *Exbucklandia*. Plastid network analysis revealed that haplotype C1 occupies a central position, connecting Clade I, Clade II, and Clade III, as well as the outgroup, suggesting that it may represent the most ancient haplotype. By integrating the geographical distribution of these haplotypes with divergence time estimates, we propose that the extant *Exbucklandia* species originated from *E. tricuspis* in tropical Vietnam, where haplotype C1 occurred. Subsequently, this species spread northward to subtropical China approximately 7 MA, leading to the formation of *E. populnea* in the western region and *E. tonkinensis* in the eastern region. Within *E. populnea*, the plastid tree indicates that subclade EII-1 diverged from its sister subclades EII-2 and EII-3 approximately 3.67 MA (Figure 4a). Geographically, subclades EII-1, EII-2, and EII-3 are distributed in the eastern, central, and western regions of the species’ range, respectively (Figure 4b). These findings support the hypothesis that *E. populnea* originated in the eastern provinces of Guizhou and Guangxi, subsequently spreading to the western province of Yunnan around 3–4 MA. Within *E. tonkinensis*, subclade EIII-1 includes four samples collected from coastal areas of Southeast China, whereas subclade EIII-2 comprises samples from inland areas. This phylogeographic pattern suggests that *E. tonkinensis* originated in Hainan and the coastal areas of Guangdong, later spreading northward into inland regions. For *E. tricuspis*, the plastid network reveals that haplotypes C2–C4 are derived from C1, indicating that *E. tricuspis* spread from Vietnam to southern Malaysia. In summary, the extant *Exbucklandia* originated from Vietnam, with *E. tricuspis* represents the most ancestral lineage. It migrated northward to subtropical China ~7 MA, where it diverged into two species: *E. populnea*, which spread further northward to Guangxi and Guizhou provinces, and *E. tonkinensis*, which spread eastward to Hainan and coastal Guangdong. In addition, *E. populnea* expanded westward to Yunnan, diverging into subclades EII-1, EII-2, and EII-3. By approximately 1 MA, *E. tonkinensis* had spread northward to the inland areas of Guangdong and neighboring regions, while *E. tricuspis* moved southward to Malaysia. The northward migration of *Exbucklandia* from tropical Vietnam to subtropical China, estimated to occur ~7 MA, coincides with the intensification of the East Asian monsoon (EAM) during the 7–99 MA period [56]. This supports the hypothesis that the establishment and intensification of the EAM significantly altered the climate of subtropical China, creating conditions conducive to the habitation of various plant species [14,57,58].

### 3.2. The Southward Expansion of E. populnea and E. tonkinensis Through Pollen Dispersal

The analysis of genetic structure based on 21 SSR loci revealed that all 50 populations originated from two gene pools: *E. populnea* predominantly possessed gene pool A, while *E. tonkinensis* harbored gene pool B. These data indicate a lack of clear structure within *E. tonkinensis* and *E. populnea*, suggesting that *Exbucklandia* has a strong capacity for long-distance pollen dispersal while fruits fall in the vicinity of maternal trees, with a very small proportion successfully dispersed over long distances. The pronounced asymmetry in pollen versus seed dispersal is common in tree plants [53,54,59] as we have mentioned above.

Further observations showed that *E. tricuspis* exhibited two scenarios: populations in South Vietnam demonstrated a mixture of gene pools A and B, while those in Malaysia were primarily composed of gene pool A. Notably, *E. tricuspis* possesses its own plastid clade I. Based on these data, it is hypothesized that both *E. populnea* and *E. tonkinensis* spread southward into Vietnam, capturing the local plastid genome. The data also suggest that *E. populnea* appeared in Vietnam earlier than *E. tonkinensis*, allowing it to further expand into Malaysia without significant admixture from gene pool B. Plastid capture is a phenomenon frequently observed in other plant species, such as Fagales [60], *Heuchera* [61], *Hieracium* [62], *Mitella* [63], and *Nothofagus* [64]. Divergence time estimation indicated that *E. tricuspis* migrated from Vietnam to Malaysia at ~1 MA, suggesting that the southward dispersal of *E. populnea* and *E. tonkinensis* occurred less than 1 MA. During this period, a decrease in temperature during the ice age may drive the southward migration of *E. populnea* and *E. tonkinensis*, a biogeographic pattern shared by other subtropical taxa, such as *Eriobotrya* [65] and *Ilex* [66].

### 3.3. Taxonomic Implication of Exbucklandia Species

Chloroplast genetic and nuclear SSR-based structure analyses consistently resolved *E. populnea* and *E. tonkinensis* as two monophyletic lineages (Figure 2a, Figure 3b,c, and Figure 4a). Their distinct morphological traits and allopatric distributions further support their recognition as distinct species. Integrative evidence from chloroplast phylogenomics, divergence dating, and dispersal modeling indicated that late Tertiary climatic cooling triggered the European extinction of *Exbucklandia*, with surviving lineages persisting in Asian tropical region. Following East Asian monsoon intensification, ancestral populations diverged into subtropical niches: *E. tonkinensis* radiated within the Sino-Japanese Floristic Region under temperate monsoonal regimes, while *E. populnea* colonized the Sino-Himalayan zone. Notably, *E. tonkinensis* exhibits consistently rhombic leaves (Figure 1f,g) with entire margins in mature individuals, contrasting sharply with the cordate–palmate leaves of *E. populnea* (Figure 1c), and the rhombic leaves progressively reduce in size along the vertical canopy gradient, which minimizes self-shading through optimized light interception. Furthermore, *E. tonkinensis* produces larger, nutrient-rich fruits and seeds, which help enhance seedling survival in low-light understories—strategies critical for competing with co-occurring evergreen broadleaved taxa [67,68].

*E. longipetala* populations present non-monophyletic in the plastid haplotype network and phylogenetic tree, interspersing with those of *E. tonkinensis* and *E. populnea*. Additionally, in SSR-based structure analyses, these populations predominantly share the gene pool *E. tonkinensis* (gene pool B), with a minor admixture from *E. populnea* (gene pool A), coupling with the absence of distinct genetic clustering. Considering their occurrences along the contact zones between the two parental species and occasionally the long-petaled trait appearing in both parental species, it is supported that these populations are hybrid offspring. In sum, *E. longipetala* populations present a morphotype rather than a natural taxon, as its circumscription relies on convergent morphological traits (long petal) without phylogenetic cohesion. Thus, *E. longipetala* should not be recognized as a distinct species, and the long petal is not a reliable trait in morphological classification in *Exbucklandia*.

*E. tricuspis* possesses a unique ancient plastid genome and morphological features (Figure 1k,l, such as tri-acuminate leaf apex in mature individuals and elongated stipules. The transient tri-acuminate leaf morphology in juvenile *E. populnea* and *E. tonkinensis*—a likely ancestral trait retained from tropical progenitors—further corroborates this scenario. However, SSR-based structure analyses reveal that *E. tricuspis* lacks a distinct nuclear (SSR) genotype; its nuclear genetic structure is characterized by an admixture between *E. populnea* and *E. tonkinensis* in Vietnam or shared with *E. populnea* in Malaysia–Indonesia region. The genetic admixture suggests ancient plastid capture by *E. populnea* during southward expansion from subtropical China, followed by *E. tonkinensis*’s subsequent hybridization with resident populations in Vietnam. This cytonuclear discordance—where plastid genomes remain distinct but nuclear genes show introgression—aligns with patterns observed in other plants [69], with a prolonged interspecific gene flow yet maintained species boundaries. Thus, we recommend recognizing *E. tricuspis* as a distinct species.

To sum up, we suggest the genus Exbucklandia comprises three distinct species: *E. tricuspis*, *E. populnea*, and *E. tonkinensis*. During Quaternary Glacial Cycles, *E. populnea* and *E. tonkinensis* dispersed southward into tropical Asia and hybridized with resident *E. tricuspis*, thereby replacing the nuclear gene pool of *E. tricuspis*. In addition, *E. populnea* and *E. tonkinensis* maintained a distribution overlap in Guangxi and Guizhou, China, where hybrid individuals were produced but a monophyletic taxon did not yet present.

### 3.4. The Genetic Diversity and Conservation of Exbucklandia Species

By comparing the expected heterozygosity (*H_E_*) and Shannon’s information index (*I*) among different populations, we conclude that the populations in Vietnam and Hainan exhibit the highest genetic diversity within their corresponding species. For example, populations such as VNSSH (*H_E_* = 0.613, *I* = 1.220), HAIDLS (*H_E_* = 0.600, *I* = 1.106) and VMBM (*H_E_* = 0.588, *I* = 1.118) significantly exceed the average genetic diversity (*H_E_* = 0.372 ± 0.118), reflecting robust allelic richness (*I* > 1.0) and balanced allele frequencies. However, genetic structure analysis reveals that the genetic diversity of these populations is primarily a result of gene flow with related species rather than in situ variation. Therefore, we recommend conservation efforts for these populations, coupled with further studies should be conducted to assess their qualification as ‘core’ germplasm populations. Currently, we recommend in addition equally selecting ‘core’ germplasm populations from non-marginal distribution areas with highest genetic diversity, such as YNBLF (*H_E_* = 0.383, *I* = 0.649) in Yunnan, China, and GDBX (*H_E_* = 0.543, *I* = 1.017) in Guangdong, China.

Previous studies demonstrated that the *F_ST_* values of Hamamelidaceae plants based on various molecular markers range from approximately 0.171 to 0.833 [70,71,72,73,74,75]. In this study, the *F_ST_* value for the genus *Exbucklandia* is 0.536 ± 0.022, indicating that the genetic diversity of this genus is at an intermediate level. This value is relatively close to that of *Disanthus cercidifolius* subsp. *longipes* (Hamamelidaceae) (*F_ST_* = 0.403), another relict tree [71].

The *F*-statistics analysis indicates that the *F_ST_* value for *E. tonkinensis* (0.432) is lower than that of others, namely *E. populnea* (0.557) and *E. tricuspis* (0.507). This discrepancy may be attributed to the impact of human activities, as the distribution area of *E. tonkinensis* is highly populated. During our field investigations in Fujian and Guangdong, we observed that numerous populations comprised fewer than 50 mature individuals. Over recent decades, local communities in these areas extensively logged *E. tonkinensis* for mountain land reclamation. As the population declines and gene flow between individuals decreases, these populations face elevated risks of inbreeding depression and potential genetic collapse. Additionally, the loss of genetic variation through random genetic drift will further diminish their evolutionary potential. Therefore, habitat restoration and population augmentation should be prioritized through urgent conservation interventions in these regions. Additionally, with high genetic differentiation (*F_ST_* > 0.5), *E. populnea* requires prioritized conservation through establishing seed banks or living gene banks to preserve their unique genetic diversity.

## 4. Materials and Methods

### 4.1. Sample Collection and DNA Extraction

Across the natural distribution of *Exbucklandia*, we collected fresh leaf samples from 23 populations of *E. populnea* and 25 populations of *E. tonkinensis* in Southern China and Vietnam, 7 populations of *E. tricuspis* in Vietnam, Malaysia, and Indonesia, and 4 populations of *E. longipetala* in Guangxi and Yunnan provinces, China (Table S6).

Fresh leaves were then dried and deposited in sealed bags with silica gel. The geographical information of the populations was recorded using a Garmin GPSMAP 62sc unit (Garmin, Shanghai, China). Voucher specimens were deposited at the Herbarium of Sun Yat-sen University (SYS). A total of 1048 individuals were sampled and selectively used in different genetic analyses. Genomic DNA of every sample was isolated from dried leaves by using the modified cetyltrimethylammonium bromide (CTAB) method [76] and purified by MicroElute DNA Clean-Up Kit D6296 (Omega Bio-Tek, Norcross, GA, USA).

Individuals from each population were used in three different molecular experiments. Due to the difficulty in extracting high-quality total DNA from fresh leaves of *Exbucklandia*, it could not be guaranteed that data from all three molecular experiments would be successfully obtained for each sample.

Based on pre-experiments, we identified two characteristics of *Exbucklandia*: (1) the extracted total DNA exhibited low purity with rapid degradation, likely due to elevated polysaccharide/protein concentrations in leaf tissue; (2) there were monomorphic chloroplast haplotypes in all sampled populations.

To balance cost efficiency, experimental feasibility, and data representativeness, we implemented the following strategies:

1. To construct a plastid tree with high support, 1 representative individual per population was selected for plastid genome sequencing, so that we could assemble complete chloroplast genome sequences to construct a plastid tree.
2. To construct a plastid haplotype network, we amplified four cpDNA fragments in 8 individuals per population, and the results showed that most populations harbored only one haplotype, suggesting that 8 individuals per population were sufficient.
3. For obtaining nuclear data, we selected populations containing ≥10 individuals to amplify with SSR primers to ensure the reliability of genetic diversity and population structure analyses.

Eventually, we successfully assembled chloroplast genomes from 34 representative individuals, amplified 4 chloroplast fragments across 56 populations (8 individuals per population), and genotyped 21 SSR loci in 50 populations (14–27 individuals per population). See Table 3 for details.

All three datasets, (1) chloroplast genomes, (2) chloroplast fragments, and (3) SSR markers, encompassed representative populations of all *Exbucklandia* species (*E. populnea*, *E. tonkinensis*, *E. tricuspis*, and *E. longipetala*). The integration of these multilocus data provides a robust framework for reconstructing the genus’ evolutionary trajectories and disentangling historical demographics from contemporary gene flow patterns.

### 4.2. Plastid Region Amplification and Haplotype Network Construction

For each population of the four *Exbucklandia* species, we randomly selected 8 individuals for PCR amplifications with primer pairs of the four chloroplast fragments (*trn*S-*psb*Z, *trn*G-*trnf*M-*rps*14, *trn*V, *rpl*32) (Tables S7 and S8). PCR amplification was performed with 2 × SanTaq PCR Mix (B8532061, Sangon Biotech Co., Ltd., Shanghai, China) following the manual instructions. PCR products were then purified and sequenced using Sanger sequencing analysis by TianYi HuiYuan Biotechnology Co., Ltd. (Wuhan, China). All the sequences were then assembled, aligned, and edited by using DNASTAR Lasergene v11.0 [77]. Haplotype network analysis of combined cpDNAs was performed with DnaSP v5.0 [78]

### 4.3. SSR Amplification, Inspection, and Diversity Analysis

Previously, we developed 24 pairs of polymorphic SSR primers for *Exbucklandia* species [50]. In this study, 21 of 24 were amplified across the 50 populations (more than 10 individuals) of the four *Exbucklandia* species (Table S9), while the remaining 3 primer pairs were excluded due to the amplification success rate (<50%). PCR amplification was performed according to the protocol described in Section 4.2. The Fragment Analyzer Automated CE System (Advanced Analytical Technologies [AATI], Ames, IA, USA) was used to inspect SSR fragments with Quant-i PicoGree dsDNA kit (1–500 bp; Invitrogen, Carlsbad, CA, USA), and PROSize v3.0 (AATI, https://www.agilent.com/en/product/automated-electrophoresis/fragment-analyzer-systems/fragment-analyzer-systems-software, access date: 16 September 2020) was used to analyze allele size. After a manual inspection and adjustment of allele size, GenAlEx v6.501 [79] was employed to calculate a range of genetic diversity indicators, including the number of observed alleles (*N_T_*), effective alleles (*N_E_*), observed heterozygosity (*H*_O_), expected heterozygosity (*H*_E_), fixation index (*F*), Shannon’s information index (*I*), and *F*-statistics analysis (*F_IS_*, *F_IT_*, *F_ST_*, *N_m_*). The genetic structure of the germplasm was analyzed using STRUCTURE software v2.3.4 [80].

### 4.4. Shallow Plastid Genome Sequencing and Assembly

For plastid genome sequencing, one sample was randomly selected for each of the 59 populations of the four *Exbucklandia* species, and high-quality total DNA material was successfully obtained for 34 samples and sent to JieRui BioScience Co., Ltd. (Guangzhou, China) for high-throughput sequencing on Hiseq X Ten (Illumina Inc.; San Diego, CA, USA) following the standard Illumina sequencing protocol with paired-end 150 bp reads, achieving a sequencing depth of 10 × coverage. The raw data were pre-processed through the program fastp with default parameters [81]. After filtering, chloroplast genomes were de novo assembled from clean reads by NOVOPlasty v2.7.2 following the manual instructions [82], selecting the cpDNA genome of *Chunia bucklandioides* (Hamamelidaceae) (NCBI: NC_041163) as a reference.

### 4.5. Phylogenetic Tree Construction and Divergence Time Estimation

For the construction of the phylogenetic tree, plastid genomes of *Rhodoleia championii* Hook. f. and *Hamamelis mollis* Oliv. were downloaded from the NCBI (National Center for Biotechnology Information) website (https://www.ncbi.nlm.nih.gov, access date: 13 July 2021) and set as an outgroup. These plastid genome sequences were aligned by MAFFT 7.475 [83], and poorly aligned positions were trimmed by TrimAl v1.1 [84] with the gappyout option. Bayesian inference (BI) and maximum likelihood (ML) methods were used to reconstruct phylogenetic trees. The BI analyses were conducted under the following conditions: starting with a random tree, 20 million generations with sampling every 1000 generations, four chains (one cold chain and three hot chains), and a burn-in of 25% trees when ASDFs (average standard deviation of split frequencies) < 0.01. For the ML analyses, IQ-TREE [85] was run using the best-fit model for each gene partition as previously selected, along with 20,000 ultrafast bootstrap replicates. FigTree v1.4.3 http://tree.bio.ed.ac.uk/software/figtree, access date: 4 October 2016) was used to visualize tree files.

The accurate application of fossil records constitutes an important step in divergence time estimation, as the selection and treatment of fossil calibration points can significantly influence the outcomes of molecular clock analyses [86]. Previous phylogenetic studies demonstrated that Subfam. Rhodoleioideae is the sister group of Subfam. Exbucklandioideae, and together, they constitute the earliest-diverging lineage within Hamamelidaceae, while the genus Hamamelis, in contrast, is representative of a late-diverging lineage within Hamamelidaceae [39,40,41,42]. Furthermore, both *Rhodoleia* and *Hamamelis* exhibit abundant fossil records with well-documented temporal constraints. Therefore, based on fossil evidence and previous studies [42,51,52,87,88], we set the following fossil calibration points in BEAST: the crown age of the *Exbucklandia*–*Rhodoleia* clade was set as 65 MA, while the age of this clade relative to *Hamamelis* was set as 100 Ma. For phylogenetic divergence time estimation, a speciation model following a Yule process was selected as the tree prior, with running for 2 million generations and sampling parameters every 1000 generations. The adequacy of sampling was assessed with Tracer v1.4 [89]. Post-run analysis’ log files indicated parameter convergence and adequate sampling (ESS values > 200). TreeAnnotator v1.4.2 [89] was used to build the maximum clade credibility tree.

## 5. Conclusions

The genus *Exbucklandia* is the sole genus within the Hamamelidaceae subfamily Exbucklandioideae and currently only comprises three species. Our results indicate that *E. longipetala* is not a distinct species due to the absence of diagnostic morphological traits and genetic monophyly. *E. tricuspis* is the earliest extant species among the current members of *Exbucklandia* and possesses its own chloroplast genome. During the Quaternary glacial period, the nuclear gene pool of *E. tricuspis* was lost due to the southward expansion of *E. populnea* and *E. tonkinensis*. Given its unique morphological characteristics and basal monophyletic position in the phylogenetic tree, we propose that *E. tricuspis* should be considered as a distinct species. Furthermore, due to habitat destruction, the genetic diversity of *E. tonkinensis* is lower than that of other species. Therefore, we recommend that conservation efforts for *E. tonkinensis* be implemented as soon as possible.

## Figures and Tables

**Table 1:** Genetic variability for the 21 SSR loci among the 50 populations of *Exbucklandia*.

Species	Pop ID	*N_T_*	*I*	*H_O_*	*H_E_*	*F*
*E. tricuspis*	IN	33	0.334	0.211	0.218	0.034
	MAFH	25	0.168	0.105	0.106	0.054
	VMBM	98	1.118	0.360	0.588	0.347
	VNBHH	40	0.379	0.244	0.238	0.044
	VNBNB	98	1.056	0.297	0.524	0.387
	VNKB	80	0.897	0.289	0.464	0.422
*E. populnea*	NP	38	0.428	0.277	0.271	−0.031
	XZ01	23	0.200	0.115	0.121	−0.010
	XZ02	34	0.393	0.222	0.241	0.051
	YNBLF	53	0.649	0.347	0.383	0.105
	YNDWS	48	0.561	0.310	0.340	0.029
	YNJTC	57	0.616	0.360	0.367	0.017
	YNMBZ	46	0.556	0.276	0.343	0.149
	YNPMZ	47	0.536	0.280	0.321	0.098
	YNQTZ	45	0.594	0.314	0.386	0.201
	YNXLS	54	0.590	0.267	0.333	0.190
	GZLB	66	0.777	0.357	0.441	0.192
	GZGHX	46	0.479	0.273	0.290	0.025
	GXBXS	49	0.592	0.290	0.362	0.157
	GXWDX	61	0.663	0.254	0.382	0.326
	GXYRT	37	0.319	0.221	0.201	−0.087
	VNSL	47	0.409	0.207	0.225	0.064
	VNNCS	41	0.380	0.226	0.220	−0.017
	VNSSH	106	1.220	0.386	0.613	0.371
	VNTPO	90	0.920	0.300	0.482	0.355
*E. longipetala*	GZLGS	39	0.429	0.324	0.278	−0.158
	GXSW	50	0.687	0.375	0.436	0.147
	HNQJD	50	0.542	0.202	0.326	0.344
	HNBMS	69	0.779	0.315	0.434	0.266
*E. tonkinensis*	GXGPS	60	0.684	0.321	0.393	0.265
	HNJQS	62	0.675	0.392	0.387	0.063
	HNMJX	78	0.946	0.369	0.522	0.339
	GDBJ	50	0.581	0.315	0.355	0.072
	GDZMP	84	0.940	0.338	0.500	0.344
	GDCDD	58	0.618	0.383	0.358	−0.096
	GDBX	90	1.017	0.476	0.543	0.129
	GDDDS	45	0.492	0.255	0.300	0.192
	GDFXC	67	0.740	0.264	0.413	0.324
	GDHSD	73	0.904	0.342	0.513	0.337
	GDNL	56	0.775	0.424	0.475	0.131
	HK	57	0.717	0.387	0.421	0.072
	HAIDLS	87	1.106	0.397	0.600	0.307
	HAIJFL	79	0.971	0.419	0.526	0.233
	HAILMS	67	0.789	0.410	0.448	0.089
	FJCBC	35	0.356	0.164	0.235	0.246
	FJDYS	47	0.427	0.193	0.250	0.229
	FJGYS	64	0.652	0.296	0.354	0.128
	FJMHS	41	0.445	0.267	0.277	0.024
	JXJPS	46	0.586	0.341	0.367	0.060
	JXWZF	61	0.763	0.427	0.455	0.081
	Total		0.649±	0.303±	0.372±	0.152±
			0.242	0.079	0.118	0.141

*N_T_*: No. of different alleles; *I*: Shannon’s information index = −1 × Sum (pi × Ln (pi)); *Ho* = observed heterozygosity = No. of *H_ETS_*/N; *H_E_* = expected heterozygosity = 1 − Sum pi^2^; *F*: fixation index = (*H_E_* − *Ho*)/*H_E_* = 1 − (*Ho*/*H_E_*).

**Table 2:** Genetic diversity determined by *F*-statistics analysis on 21 SSR loci.

	*F_IS_*	*F_IT_*	*F_ST_*	*N_M_*
*E. populnea*	0.126 ± 0.026	0.609 ± 0.041	0.557 ± 0.04	0.248 ± 0.035
*E. tonkinensis*	0.18 ± 0.038	0.533 ± 0.031	0.432 ± 0.023	0.367 ± 0.037
*E. tricuspis*	0.283 ± 0.07	0.643 ± 0.045	0.507 ± 0.032	0.283 ± 0.036
*E. longipetala*	0.116 ± 0.063	0.584 ± 0.035	0.503 ± 0.042	0.321 ± 0.048
Genus *Exbucklandia*	0.189 ± 0.033	0.621 ± 0.027	0.536 ± 0.022	0.232 ± 0.02

*F_IS_* (fixation index total): intraspecific inbreeding coefficient; *F_IT_* (fixation index total): population inbreeding coefficient; *F_ST_* (fixation index of subpopulations relative to the total population): fixed coefficient; *N_M_* (number of migrants per generation): gene flow.

**Table 3:** Fifty-nine *Exbucklandia* populations collected and used for molecular experiments in this study.

Pop ID	Country	Research Contents (Number of Individuals)			Pop ID	Country	Research Contents (Number of Individuals)		
		A *	B **	C ***			A *	B **	C ***
***E. tonkinensis***					***E. populnea***				
FJMHS	China		★(8)	●(15)	XZ01	China		★(8)	●(22)
FJGYS	China		★(8)	●(23)	XZ02	China		★(8)	●(18)
FJCBC	China		★(8)	●(27)	YNQTZ	China	▲(1)	★(8)	●(20)
FJDYS	China		★(8)	●(17)	YNXLS	China	▲(1)	★(8)	●(18)
GDBJ	China		★(8)	●(24)	YNDWS	China	▲(1)	★(8)	●(16)
GDHSD	China	▲(1)	★(8)	●(16)	YNMBZ	China	▲(1)	★(8)	●(19)
GDDDS	China	▲(1)	★(8)	●(20)	YNJTC	China	▲(1)	★(8)	●(23)
GDWZS	China		★(8)		YNQLD	China	▲(1)	★(8)	
GDNL	China	▲(1)	★(8)	●(19)	YNGLG	China	▲(1)	★(8)	
GDCDD	China		★(8)	●(19)	YNYFS	China		★(8)	
GDZMP	China	▲(1)	★(8)	●(20)	YNPMZ	China	▲(1)	★(8)	●(23)
GDFXC	China		★(8)	●(24)	YNHQZ	China		★(8)	
GDBX	China		★(8)	●(24)	YNBLF	China	▲(1)	★(8)	●(21)
GXLJ	China	▲(1)			NP	Nepal		★(8)	●(16)
GXGPS	China			●(16)	VNTPO	Vietnam		★(8)	●(24)
HAIJFL	China	▲(1)	★(8)	●(19)	VNNCS	Vietnam		★(8)	●(20)
HAIJX	China	▲(1)	★(8)		VNSSH	Vietnam	▲(1)	★(8)	●(19)
HAILMS	China	▲(1)	★(8)	●(21)	VNSL	Vietnam	▲(1)	★(8)	●(20)
HAIDLS	China	▲(1)	★(8)	●(15)	***E. tricuspis***				
HK	China	▲(1)	★(8)	●(15)	IN	Indonesia	▲(1)	★(8)	●(16)
HNMJX	China		★(8)	●(24)	MATR	Malaysia	▲(1)	★(8)	
HNQYF	China	▲(1)			MAFH	Malaysia	▲(1)	★(8)	●(14)
HNJQS	China		★(8)	●(25)	VNKB	Vietnam		★(8)	●(16)
JXWZF	China	▲(1)	★(8)	●(20)	VNBNB	Vietnam	▲(1)	★(8)	●(21)
JXJPS	China		★(8)	●(19)	VNBHH	Vietnam	▲(1)	★(8)	●(15)
***E. populnea***					VMBM	Vietnam		★(8)	●(25)
GXYRT	China		★(8)	●(20)	***E. longipetala***				
GXBXS	China	▲(1)	★(8)	●(20)	GXSW	China	▲(1)	★(8)	●(20)
GXWDX	China	▲(1)	★(8)	●(21)	GZLGS	China	▲(1)	★(8)	●(20)
GZLB	China		★(8)	●(22)	HNQJD	China	▲(1)	★(8)	●(20)
GZGHX	China	▲(1)	★(8)	●(15)	HNBMS	China		★(8)	●(24)

* Genome skimming: Assembled chloroplast genomes and ribosomal cistrons from 1 individual per population for phylogenetic reconstruction. ** Sanger sequencing: Analyzed 4 cpDNA fragments across 8 individuals per population to construct haplotype networks. *** SSR polymorphism analysis: Investigated population structure using 21 loci.

## Data Availability

All data are publicly available in the article and Supplementary Materials.

## Supplementary Materials

The following supporting information can be downloaded at https://www.mdpi.com/article/10.3390/plants14071061/s1, Table S1. Twenty-one haplotypes of combined chloroplast fragments in 56 *Exbucklandia* populations; Table S2. Variable sites of the aligned sequences (5′-3′) in 21 haplotypes of *Exbucklandia* (2345 bp without outgroup); Table S3. Genetic diversity at the 21 microsatellite loci of *Exbucklandia*; Table S4. Result of *F*-statistic on locus; Table S5. Length for each partition in chloroplast genomes of 34 *Exbucklandia* individuals; Figure S1. Phylogenetic tree of 34 *Exbucklandia* individuals with 29 outgroups based on BI; Figure S2. Phylogenetic tree of 34 *Exbucklandia* individuals with *Rhodoleia championii* on BI; Figure S3. Phylogenetic tree of 34 *Exbucklandia* individuals with *Rhodoleia championii* on ML; Table S6. Voucher information of 59 *Exbucklandia* populations in this study; Table S7. Development of chloroplast primers; Table S8. Chloroplast primers used in this study; Table S9. Characteristics of 21 SSR loci used in this research.
