# Supplementary material for: Multiple Dataset-Based Insights into the Phylogeny and Phylogeography of the Genus Exbucklandia (Hamamelidaceae): Additional Evidence on the Evolutionary History of Tropical Plants
Source: Plants (Basel). 2025 Mar 29;14(7):1061. doi: 10.3390/plants14071061 (PMC11991608; doi:10.3390/plants14071061)
Supplement: Supplementary file 1 [file plants-14-01061-s001.zip › plants-3469960-supplementary.pdf]

Supplementary Table S1. 21 Haplotypes of combined chloroplast fragments in 56 *Exbucklandia* populations

Haplotype	Population ID
C1	VNBNB、VMBM、VNBHH、VNKB
C2	IN
C3	MATR
C4	MAFH
C5	VNSL
C6	GDTGZ
C7	HAIJFL、HAILMS、GDBX、FJDYS、HAIDLS、HAIJX、JXJPS
C8	FJMHS
C9	HK
C10	JXWZF、HNMJX、HNJQS、FJGYS、GDHSD、GDFXC、FJCBC、GDBJ、GDCDD、GDDDS、GDWZS、GDNL、HNBMS、HNQJD
C11	YNMBZ、YNDWS、YNJTC、YNQTZ、YNXLS
C12	VNNCS
C12	VNTPO、GWDXJ、GZLB、GXBXS、GXYRT
C13	XZ01、YNPMZ、YNGLG2、YNPMZ、YNQLD
C14	VNSSH
C15	GZGHX
C16	YNGLG1、NP
C18	YNBLF
C19	XZ02
C20	GZLGS
C21	GXSW

Supplementary Table S2. Variable sites of the aligned sequences (5'-3') in 21 haplotypes of *Exbucklandia* (2345bp without outgroup)

Haplotype	Variable position																								
	*trn*S-*psb*Z (1—641)						*trn*G-*trnf*M-*rps*14 (642—1250)							*trn*V (1251—1844)							*rpl*32 (1845—2345)				
	20	159	285	286	305	471	643	732	771	1033	1068	1159	1233	1254	1272	1525	1681	1712	1753	1873	1892	1955	2171	2211	2310
C1	T	T	T	A	A	A	T	A	T	T	G	T	A	A	A	G	C	G	T	A	T	A	T	T	T
C2	T	T	T	A	A	A	T	A	T	T	G	T	A	A	A	G	C	G	C	A	T	A	T	T	T
C3	T	T	T	A	A	A	T	A	T	T	G	T	A	A	A	G	C	G	C	A	T	C	T	T	T
C4	T	G	T	A	A	A	T	A	T	T	G	T	A	A	A	G	C	G	C	A	T	A	T	T	T
C5	T	T	T	A	A	C	T	A	T	T	G	T	A	A	A	A	C	G	T	A	C	A	T	G	T
C6	T	T	T	A	A	A	C	A	T	T	G	T	G	G	G	G	C	G	T	A	C	A	T	G	T
C7	T	T	T	A	G	A	T	A	C	T	G	T	A	G	G	G	C	G	T	A	C	A	T	G	T
C8	C	T	T	A	G	A	T	A	C	T	G	T	A	G	G	G	C	G	T	A	C	A	T	G	T
C9	T	T	T	C	G	A	T	A	C	T	G	T	A	G	G	G	C	G	T	A	C	A	T	G	T
C10	T	T	T	A	G	A	T	G	C	T	G	T	A	G	G	G	C	G	T	A	C	A	T	G	T
C11	T	T	T	A	A	C	T	A	T	T	G	T	A	A	A	A	C	G	T	C	T	A	T	T	T
C12	T	T	T	A	G	A	T	A	T	T	G	T	A	A	A	A	C	G	T	C	T	A	C	T	T
C13	T	T	G	A	A	A	T	A	T	T	G	T	A	A	A	A	C	G	T	C	T	A	T	T	G
C14	T	T	T	A	A	A	C	A	T	T	G	T	G	A	A	A	C	G	T	C	T	A	C	T	T
C15	T	T	T	A	A	C	T	A	T	G	A	G	A	A	A	A	C	G	T	C	T	A	T	T	T
C16	T	T	T	A	A	A	T	A	T	T	G	T	A	A	A	A	C	G	T	C	T	A	T	T	G
C17	T	T	T	A	A	A	T	A	T	T	G	T	A	A	A	A	C	G	T	C	T	A	C	T	T
C18	T	T	T	A	A	A	C	A	T	T	G	T	G	A	A	A	G	G	T	C	T	A	C	T	T
C19	T	T	T	A	A	A	C	A	T	T	G	T	G	A	A	A	G	A	T	C	T	A	C	T	T
C20	T	T	G	A	A	A	T	A	T	T	G	T	A	A	A	A	C	G	T	C	T	A	T	T	T
C21	T	T	T	A	A	A	T	A	C	T	G	T	A	G	G	G	C	G	T	A	C	A	T	G	T

* Note:

** Haplotype C1:

TATTGGGATCAGGTCATCCTTAAAAAGTATTCCTTTTTCCTTTCTTTTTTTTTCAAATCTATATCTAATCCATTCTTTTTCTGGCTCGGCTAGGTGGGATAGCCGAGCCATTCCCCTTTATGATACCGGGCC
GGACAAAACCAATAAAGAAAGAAATCTATTCACCGAGCAAAAGGAGAGAGAGGGATTCGAACCCTCGATAGTTCTGAACAAAGAACTATACCGGTTTTCAAGACCGGAGCTATCAACCACTCGGC
CATCTCTCCCAAAGACAATTTCTATTTTATTTTTTATTCCCCCGAATGGAACATAGCCATATGAGTTCATACCATCAGTATCTGTAGAAACAGGTATGAATCTATGGTCGATCTATCTATCTGTATATATAG
ATAGATGCAAGATCCAGCATGTCCATTTGTGAAGTAAAAAAGATTCCCCTCGACCCCATGTCCGAATAAAATGGTAAAAAAAAGGGGGGTGATAAGTCATATAGAATCAATGGATTCATGGTAAAAA
AAATCCCTCCATGATGCATTTTATTACAATTTTTCGATTGATAGAGGGATCAAATGGTATAGTTCTTTTGTTGGTAGCTTGGAGGATTAGAAGCATGGCTATTGCTTTCCAATTGGCTGTTTTTGTTTCTC
TTTGCCAGGAGAAGACGCGGGTTCGATTCCCGCTATCCGCCTAGGGTAAAGTAATGTATTTAATTTAATATGATAAATGATAAAAGATTCGGTATAGTTGACCGTGATAGTGGAGTGGCTCTACCCTCC
CCCTTCTTTTCCTCTCACCTCAAAAAGAAATATAGTAATTAATTACTAGTTAACAGAGTCGAACCTAATTTTTTTTTACAAAAAAAAATGTTGCGGAGACAGGATTTGAACCCGTGACCTCAAGGTTATG
AGCCTTGCGAGCTACCAAGCTGCTCTACCCCGCGCTGAAGAGAAGAACTGGGAACTAATGGACAAACAAGGATTGAATGCGCCCCTCTACCATATCTGTACAAATAGAATAGCCCATTTATACAGAA
TGGTAAAGGGGGCCCCTCTATGATCGATGATTATAGAAATAAATAATGAAGGGATATTTTAATCCTTACCAACTTGATCTTGTTGCCCCTGGCAACAAACATGCATGAACCATTTCACGAAGTATGTGT
CCGGATAGCCCAAAGTCTCGATAGTTAGCTCTGGGTCTTCCGGTCGAAAAACAACGTCGATGAAGACGTGTAGGTGCACTATTACGTGGATTGGGTAGGTAGACCAAAGAGGTATCACTAATTCTTT

GATTGTGGACAGGAAAATTCATATGTAATTCATCTACGAAGATTAATGAAGAAAAATGGGTTTGTTTATCCGAGATTGGAAAAATTTCGATTGGATCCATTGAAATGCGTTTTTGTTTTCAGTTTTATGT
TCTGCTCTGGACGATCCCCGTGAGCGAGCTTATGGGAATGATTTTATTTCGATGAACCAAGCAACCGACCAGTTACAAACAACAAACAATACAATAATGAGGAAATGAAAACTATACAATTTTTGTAT
TTGAATCATTTTATTTCATTTAGGGCTATACGGACTCGAACCGTAGACCTTCTCGGTAAAACAGATCAAACTTATTATTATCAAAATGATTCGAACTGTTTCAAAGACCCAACATGCATTTTTTTTGCAT
TGGGCTCTTTCATTAACTGATATAAATATCAGCTAGTCCACCATATTTTTTCTTGACAGAAAGATAAGGAGATGGCTCCATGCGCTCTGATTCATTATTTGGATTCTGATCCAGGAGCACTACCAAAGTG
TTTCAAAGAAGGGTTATCTTGACGTAGGTCTGCCTTTGTCATTCATTTTATATTATAAACGAGATTAACCCACTTTTCGAGTCAGCCCTATTCAGATTATTCTAACGTTTGATTATTTATTTGTATTTGTCG
GCACAAAAAAAACTTTTAGAATTCCCGGTAGAAAGAGATTTCGCTAATGAGAAAGCTTTTAACGCTGCCCAATATCCCTTCCTTTTCCAAAATTTTTTACGAATACGCTTTTTTGACATAGAAGTACGTT
TTTTTGGAACTGCCATTCAAAAATTAAGAGATTACTCATTGCTATAGTTGGATGTGAAAGACATCTATTGTTCAAAACGAATCCCTCTTTACTATTCATTATCTATCTATTTATTTTATAGATGATACTCCC
TTCATAAAAAAAGAAATATAGATATGTGAAAATCGCATAAAATATTACTAGGGACAGATAAAAAAAGTTGTTTTTTTTATTTCTATTCCATACAATATCCGGACGAAAAAATAATTCGTACTGATTGGTGC
CCCTGGTATTCAAATC

***Outgroup (*Rhodoleia championii*):
TATTGGGATCAGGTCATCCTTAAAAAGTATTCCTTCTTCCTTTCTTTTTTTTTTTTTTTTCAAATCTATATCTAATCTATTCTTTTTCTGGCTCGGCTAGGTGGGATAGCCGAGCCATTCCCCTTTATGTTTAT
GATACCGGGCCGGGCAAAACCAATAAAGAAAGAAATCTATTCACCGAGCAAAAGGAGAGAGAGGGATTCGAACCCTCGATAGTTCTGAACAAAGAACTATACCGGTTTTCAAGACCGGAGCTATC
AACCACTCGGCCATCTCTCCCAAAGACAATTTCTATTTTATTTTTTATTCCCCCGAATAGAACATAGCCATATGAGTTCATACCATCAGTATCTGTAGAAAAATATCAAGTATGAATCTATGGTCGATCTA
TCTATCTGTATATATAGATAGATGCAAGATCCAGCATGTCCATTTGTGAAGTAAAAAAGATTTCCCTCGACCCCATGTCCGAATAAAGTGGTAAAAAAAAAATGGTGATAAGTCATATAGAATCACTGGA
TTCATGGTAAAATCCCTCCATGATGCATTTTATTACAATTTTTCGACTGATAGAGGGATCAAATGGTATAGTTCTTTTGTTGGTAGCTTGGAGGATTAGAAGCATGACTATTGCTTTCCAATTGGCTGTTT
TTGTTTCTCTTTGCCAAGGAGAAGACGCGGGTTCGATTCCCGCTATCCGCCTAGGGTAAAGGAATGTATTTAATTTAATATGATAAATGATAAAAGATTCGGTATAGTTGACCGTGATAGTGGAGTGGC
TCTATCTTCCCCCTTCTTTTCCTCTCACCTCAAAAAGAAATATAGTAATTAATTACTAGTTAACAGAGTCGAACCTAAATTTTTTTTACAAAAAAAAATGTTGCGGAGACAGGATTTGAACCCGTGACCT
CAAGGTTATGAGCCTTGCGAGCTACCAAGCTGCTCTACCCCGCGCTGAAGAGAAGAACTGGGAACTAATGGACAAACAAGGATTGAATGCGCCCCTCTACCATATCTGTACAAATAGAATAGCCCAT
TTATACAGAATGGTAAAGGGGGCCCCTCTATGATCGATGATCATAGAAATAAATAATGAAGGGATATTTTAATCCTTACCAACTTGATCTTGTTGCCCCTGGCAACAAACATGCATGAACCATTTCACG
AAGTATGTGTCCGGATAGCCCAAAGTCTCGATAGTTAGCTCTGGGTCTTCCGGTCAAAAAACAACGTCGATGAAGACGTGTAGGTGCACTATTACGTGGATTAAGTAGGTAAACCAAAGAAGTATCA
CTAATTCTTTGATTGTGGATAGGAAAAGAGGAAAATTCATATATAATTCATCTACGAAGATTAATGAGGAAAAATAGGTTTGTGTATTCGAGATTGGAAAAATATCGATTGGATCCATTGAAATGCGTTT
TTGTTTTCCGTTTTATGCTCTGCTCTGGACGATCCCCGTGAGCGAGCTTATGGGAATGATTTTCTTTCGATGAACCAAGCAACCGGCCAGTTACAAACAACAAACAATACAATAATGAGGAAATGAAA
ACTATACAATTTTTGTATTTGAATCATTTTATTTCATTTAGGGCTATAGGGCTATACGGACTCGAACCGTAGACCTTCTCGGTAAAACAGATCAAACTTATTATTATCAAAATGATTCGAACTGTTTCAAA
GACCCAACATGCGTTTTTTTTGCATTGGGCTCTTTCATTAACTGATATAAATATCAGTTAGTCCACCATATTTTTTCTTGACAGAAAGATAAGGAGATGGCTCCATGCGCTCTGATTCATTATTTGGATTC
TGATCCAGGAGCACTACCAAAGTGTTTCAAAGAAGGGTTATCTTGACGTAGGTCTGCCTTTGTCATTTTTTATTATATTATAAACAAAATTAACCCACTTTTCGAGTCAGTCTGATTCAGGTTATTCTAA
CGTTTGATTATTTATTTGTATTTGTCGCTACAAAAAAACTTTTTGAATTCCCGGTAGAAAGAGATTTCGCTAATGAAAAAGCTTTTAACGCTGCCCAATATCCCTTCCTTTTCCAAAACTTTTTACGAAT
ACGCTTTTTTGACATAGAAGTACGTTTTTTTGGAACTACCATTCAAAAATTAAGAGATTACTCATTACTATAGTTGGATGTGAAAGACATCTATTGTTCAAATGTTCAAAACGAATCCTTCTTTACTATT
CATTATCTATCTATTTATTTTATAGATGATACTCCCTTCATAAAAAATCAATATAGATATGTGAAAATCGCATAAAATATTACTAAGGACAGATAAAAAAATTGTGTTTTTTTTTGTTTCTATTAATTGGTCA
AGCTCGAAGAGAGAATACACCTAATTTTAATTTTCAAATTCGAATGAGACTAGTAATC

Supplementary Table S3. Genetic diversity at the 21 microsatellite loci of *Exbucklandia*

Locus	N_T	N_E	I	H_O	H_E	F_{IS}	F_{IT}	F_{ST}	N_M
EX301	18	1.665	0.514	0.236	0.297	0.204	0.736	0.668	0.124
EX302	16	2.096	0.763	0.355	0.432	0.179	0.594	0.505	0.245
EX327	12	2.000	0.724	0.316	0.415	0.239	0.632	0.516	0.234
EX336	10	1.587	0.434	0.205	0.271	0.242	0.701	0.605	0.163
EX337	10	2.033	0.746	0.306	0.434	0.295	0.570	0.390	0.391
EX346	12	2.003	0.720	0.313	0.409	0.235	0.626	0.511	0.240
EX353	11	1.748	0.581	0.072	0.329	0.278	0.734	0.631	0.146
EX357	17	2.455	0.945	0.423	0.532	0.205	0.532	0.412	0.357
EX358	13	2.468	0.836	0.286	0.440	0.351	0.669	0.490	0.260
EX359	15	2.033	0.697	0.254	0.397	0.360	0.711	0.549	0.206
EX365	16	2.259	0.815	0.356	0.446	0.201	0.588	0.484	0.267
EX378	13	1.644	0.458	0.171	0.248	0.311	0.785	0.688	0.113
EX379	12	1.398	0.390	0.205	0.229	0.103	0.762	0.735	0.090
EX403	10	1.699	0.601	0.264	0.350	0.245	0.691	0.591	0.173
EX406	7	1.312	0.336	0.131	0.198	0.339	0.704	0.553	0.202
EX410	6	1.855	0.603	0.481	0.375	-0.281	0.349	0.492	0.258
EX506	7	1.771	0.599	0.385	0.365	-0.057	0.415	0.447	0.310
EX608	12	2.424	0.923	0.418	0.518	0.192	0.500	0.381	0.406
EX609	8	1.516	0.429	0.195	0.251	0.223	0.751	0.679	0.118
EX610	10	2.032	0.761	0.377	0.441	0.147	0.546	0.468	0.285
EX6511	9	1.960	0.755	0.464	0.445	-0.042	0.438	0.460	0.293
Total		1.903±	0.649±	0.296±	0.372±	0.189±	0.621±	0.536±	0.232±
		0.323	0.17	0.107	0.092	0.033	0.027	0.022	0.020

Supplementary Table S4. Result of *F*-statics on locus.

	Exbucklandia				***E. populnea***				***E. tonkinensis***				***E. tricuspis***				***E. longipetala***			
**Locus**	F_{IS}	F_{IT}	F_{ST}	N_M	F_{IS}	F_{IT}	F_{ST}	N_M	F_{IS}	F_{IT}	F_{ST}	N_M	F_{IS}	F_{IT}	F_{ST}	N_M	F_{IS}	F_{IT}	F_{ST}	N_M
**EX301**	0.205	0.736	0.668	0.124	0.156	0.688	0.630	0.147	0.268	0.614	0.473	0.279	0.143	0.929	0.917	0.023	-0.257	0.767	0.815	0.057
**EX302**	0.177	0.593	0.505	0.245	0.062	0.397	0.357	0.450	0.122	0.549	0.486	0.264	0.106	0.497	0.437	0.322	0.571	0.788	0.505	0.245
**EX327**	0.241	0.632	0.516	0.235	0.090	0.641	0.606	0.163	0.451	0.657	0.375	0.417	0.030	0.507	0.492	0.258	-0.067	0.343	0.384	0.401
**EX336**	0.242	0.701	0.605	0.163	0.214	0.587	0.475	0.277	0.182	0.587	0.495	0.255	0.641	0.895	0.709	0.103	-0.042	0.829	0.836	0.049
**EX337**	0.295	0.570	0.390	0.390	0.223	0.564	0.439	0.319	0.337	0.510	0.261	0.708	0.345	0.498	0.233	0.822	0.081	0.402	0.349	0.466
**EX346**	0.234	0.625	0.510	0.240	0.054	0.524	0.497	0.253	0.439	0.680	0.429	0.333	0.410	0.679	0.456	0.298	-0.048	0.401	0.428	0.334
**EX353**	0.276	0.733	0.631	0.146	0.109	0.781	0.754	0.082	0.168	0.657	0.588	0.175	0.578	0.789	0.501	0.249	0.223	0.600	0.485	0.266
**EX357**	0.205	0.532	0.412	0.357	0.059	0.357	0.317	0.539	0.124	0.438	0.358	0.448	0.526	0.751	0.476	0.275	0.164	0.421	0.307	0.564
**EX358**	0.350	0.668	0.490	0.260	0.172	0.553	0.460	0.294	0.361	0.704	0.536	0.216	0.371	0.605	0.371	0.423	0.481	0.637	0.299	0.585
**EX359**	0.363	0.713	0.549	0.206	0.306	0.688	0.550	0.205	0.307	0.670	0.524	0.227	0.536	0.707	0.370	0.426	0.363	0.570	0.325	0.519
**EX365**	0.201	0.587	0.483	0.267	0.094	0.416	0.355	0.454	0.116	0.512	0.448	0.308	0.566	0.795	0.528	0.224	0.225	0.464	0.308	0.561
**EX378**	0.307	0.784	0.689	0.113	-0.052	0.843	0.851	0.044	0.266	0.594	0.447	0.309	0.489	0.769	0.548	0.206	0.316	0.764	0.655	0.132
**EX379**	0.103	0.762	0.735	0.090	0.014	0.813	0.810	0.059	0.146	0.705	0.655	0.132	-0.049	0.479	0.503	0.247	0.257	0.846	0.793	0.065
**EX403**	0.244	0.691	0.591	0.173	0.189	0.786	0.736	0.090	0.191	0.570	0.469	0.283	0.304	0.669	0.525	0.226	0.068	0.612	0.584	0.178
**EX406**	0.346	0.708	0.553	0.202	0.226	0.894	0.863	0.040	0.295	0.550	0.362	0.440	0.661	0.852	0.563	0.194	-0.007	0.557	0.560	0.196
**EX410**	-0.281	0.349	0.492	0.258	-0.231	0.182	0.335	0.496	-0.185	0.421	0.511	0.239	-0.489	0.160	0.436	0.324	-0.774	0.598	0.773	0.073
**EX506**	-0.057	0.416	0.448	0.308	0.162	0.551	0.465	0.288	-0.084	0.281	0.337	0.492	-0.421	0.168	0.415	0.353	0.129	0.574	0.511	0.239
**EX608**	0.190	0.499	0.381	0.406	0.155	0.446	0.344	0.476	0.183	0.404	0.270	0.675	0.290	0.533	0.343	0.479	0.452	0.699	0.451	0.304
**EX609**	0.222	0.750	0.679	0.118	0.288	0.871	0.819	0.055	0.132	0.528	0.456	0.298	0.497	0.762	0.527	0.224	0.033	0.666	0.655	0.132
**EX610**	0.147	0.546	0.468	0.284	0.171	0.628	0.551	0.203	0.160	0.418	0.307	0.565	0.397	0.779	0.634	0.144	0.127	0.394	0.306	0.566
**EX6511**	-0.042	0.438	0.460	0.293	0.179	0.576	0.484	0.267	-0.192	0.139	0.278	0.650	0.001	0.669	0.669	0.124	0.132	0.337	0.235	0.813
**Mean**	0.189	0.621	0.536	0.232	0.126	0.609	0.557	0.248	0.180	0.533	0.432	0.367	0.283	0.643	0.507	0.283	0.116	0.584	0.503	0.321
**SE**	0.033	0.027	0.022	0.020	0.026	0.041	0.040	0.035	0.038	0.031	0.023	0.037	0.070	0.045	0.032	0.036	0.063	0.035	0.042	0.048

Supplementary Table S5. Length for each partition in chloroplast genomes of 34 *Exbucklandia* individuals

Sample ID	Pop ID	Species	Length (bp)	LSC (bp)	IR (bp)	SSC (bp)	GenBank accession numbers
ZWY401	GZLGS	*E. longipetala*	160756	89002	26422	18910	PV132461
ZWY498	GXSW	*E. longipetala*	160780	89038	26421	18892	PV132462
QJD1	HNQJD	*E. longipetala*	160814	89060	26429	18896	PV132460
16660	YNBLF	*E. populnea*	160554	88797	26422	18913	PV132467
ZWY785	YNMBZ	*E. populnea*	160657	88898	26422	18915	PV132475
62316	GXBXS	*E. populnea*	160723	88972	26422	18907	PV132469
GX209	GXWDX	*E. populnea*	160723	88972	26422	18907	PV132471
ZWY445	GZGHX	*E. populnea*	160723	88972	26422	18892	PV132474
VNSL15	VNSL	*E. populnea*	160742	88988	26422	18910	PV132472
VNSP03	VNSSH	*E. populnea*	160742	88988	26422	18910	PV132473
15331	YNQTZ	*E. populnea*	160744	88989	26422	18911	PV132463
16001	YNJTC	*E. populnea*	160744	88989	26422	18911	PV132465
15342	YNDWS	*E. populnea*	160747	88994	26422	18902	PV132464
16753	YNXLS	*E. populnea*	160753	88994	26422	18915	PV132468
16656	YNPMZ	*E. populnea*	160806	89048	26422	18907	PV132466
ZWY866	YNQLD	*E. populnea*	160821	89069	26422	18901	PV132476
87800	YNGLG	*E. populnea*	160905	89149	26422	18912	PV132470
JDL102	HAIJFL	*E. tonkinensis*	160673	88925	26429	18890	PV132487
GD164	GDZMP	*E. tonkinensis*	160710	88931	26429	18921	PV132477
H01	HAILMS	*E. tonkinensis*	160723	88960	26437	18904	PV132480
J101	JXWZF	*E. tonkinensis*	160727	88974	26429	18900	PV132486
GDW015	GDNL	*E. tonkinensis*	160753	88993	26429	18909	PV132478
GX104	GXLJ	*E. tonkinensis*	160767	89017	26429	18897	PV132479
HK013	HK	*E. tonkinensis*	160774	89028	26429	18893	PV132483
H37	HAIDLS	*E. tonkinensis*	160790	89032	26429	18900	PV132482
HN50	HNQYF	*E. tonkinensis*	160790	89036	26429	18896	PV132484
HSD1	GDHSD	*E. tonkinensis*	160790	89036	26429	18896	PV132485
LZ07	GDDDS	*E. tonkinensis*	160804	89036	26429	18917	PV132488
H102	HAIJX	*E. tonkinensis*	160825	89073	26429	18901	PV132481
VN103	VNBNB	*E. tricuspis*	160728	88982	26424	18893	PV132458
I12	IN	*E. tricuspis*	160760	89016	26424	18896	PV132455
VN401	VNBHH	*E. tricuspis*	160762	89016	26424	18898	PV132459
MA39	MATR	*E. tricuspis*	160771	89022	26424	18904	PV132457
MA07	MAFH	*E. tricuspis*	160772	89029	26424	18890	PV132456

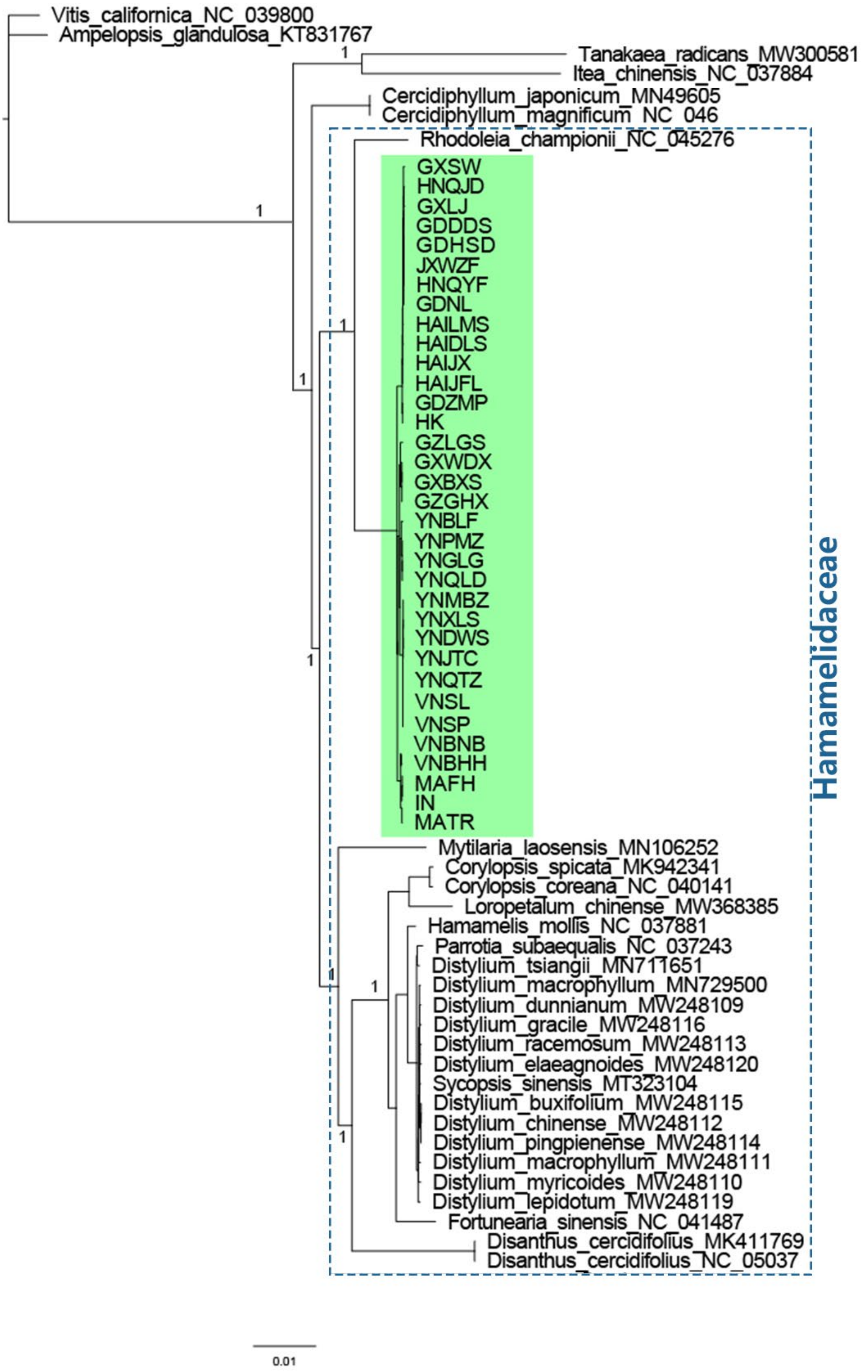

Supplementary Figure S1 Phylogenetic tree of 34 *Exbucklandia* individuals with 29 outgroups based on BI. Posterior probabilities (PP) ≥0.95 are shown above branches.

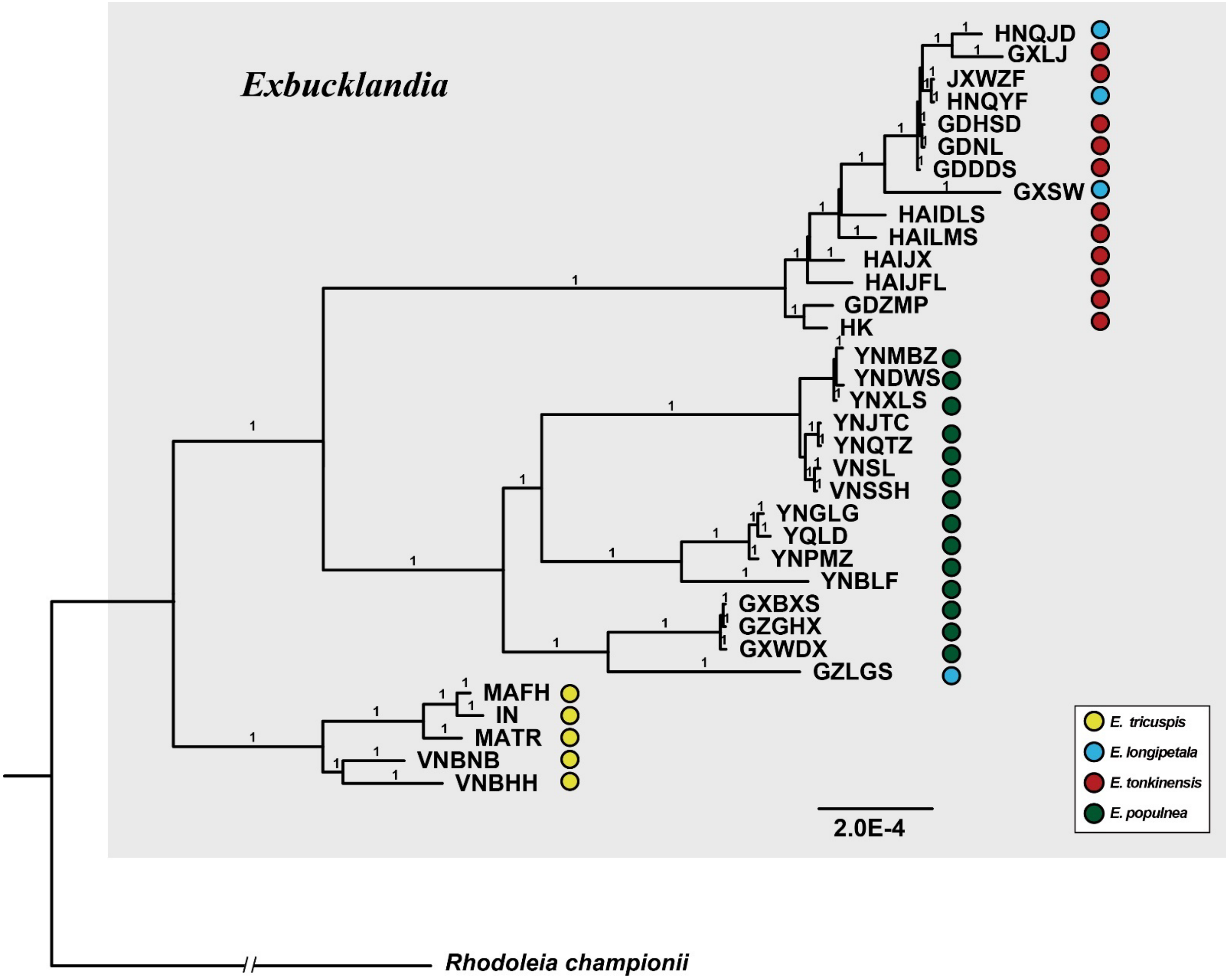

Supplementary Figure S2 Phylogenetic tree of 34 *Exbucklandia* individuals with *Rhodoleia championii* based on BI. Posterior probabilities (PP) ≥0.95 are shown above branches.

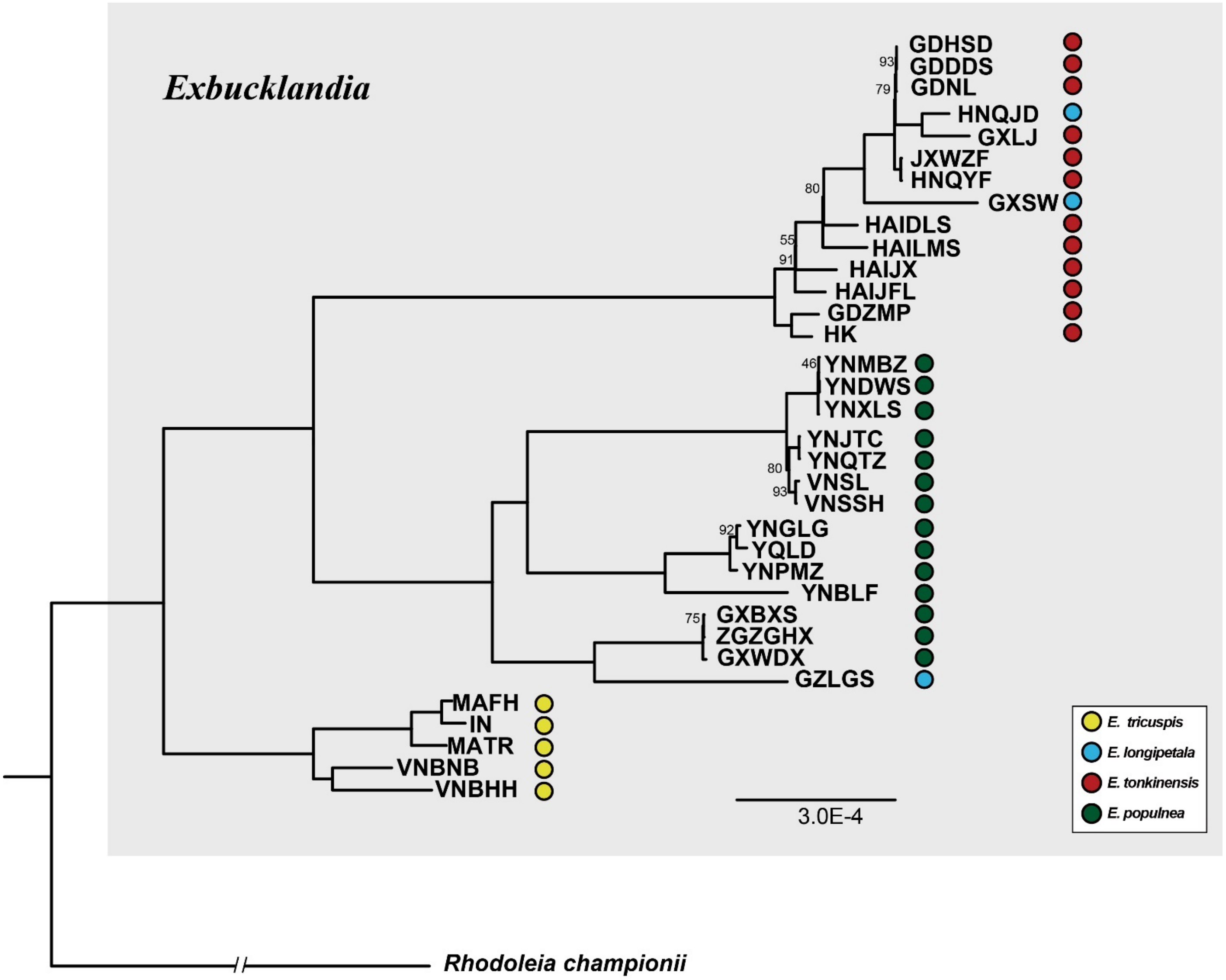

Supplementary Figure S3 Phylogenetic tree of 34 Exbucklandia individuals with Rhodoleia championii based on ML. Bootstrap values (BS) are shown above branches. Values > 95% are omitted for clarity.

Supplementary Table S6. Voucher information of 59 *Exbucklandia* populations in this study.

Pop ID	Specie	Country	Location	Longitude and latitude	Elevation	Collectors	Collection number	Voucher specimens
FJMHS	*E. tonkinensis*	China	Fujian Province: Meihua Mountain National Nature Reserve	116.8573 E; 25.3360 N	1200m	C. Y. Huang; Y. R. Liu; F. Ye	HCY180702	SYS00237050
FJGYS	*E. tonkinensis*	China	Fujian Province: Gongyashan Mountain	117.4084 E; 24.922 N	979m	C. Y. Huang; Y. R. Liu; F. Ye	HCY180701	SYS00237051
FJCBC	*E. tonkinensis*	China	Fujian Province: Cheben Village, Zhangpu County	117.4605 E; 24.2568 N	705m	C. Y. Huang; Y. R. Liu; F. Ye	HCY180703	SYS00237052
FJDYS	*E. tonkinensis*	China	Fujian Province: Daiyun Mountain National Nature Reserve	118.2280 E; 25.6350 N	954m	C. Y. Huang; Y. R. Liu; F. Ye	HCY180608	SYS00237054
GDBJ	*E. tonkinensis*	China	Guangdong Province: Xianjiadong Reservoir, Yangchun City	111.4219 E; 21.8588 N	685m	Q. Fan	13540	
GDHSD	*E. tonkinensis*	China	Guangdong Province: Heishiding Nature Reserve	111.8851 E; 23.4437 N	213m	Q. Fan	13484	
GDDDS	*E. tonkinensis*	China	Guangdong Province: Dadong Mountain, Qingyuan City	112.7494 E; 24.9199 N	901m	C. Y. Huang; Y. R. Liu; F. Ye	HCY1733	
GDWZS	*E. tonkinensis*	China	Guangdong Province: Wuzhishan Mountain, Conghua City	113.7622 E; 23.7075 N	914m	C. Y. Huang; Y. R. Liu; F. Ye	HCY1734	
GDNL	*E. tonkinensis*	China	Guangdong Province: Nanling Mountains, Ruyuan County	112.9549 E; 24.9261 N	1432m	H. W. Wang	W18002	
GDCDD	*E. tonkinensis*	China	Guangdong Province: ChuanDi Ding Mountain, Shaoguan City	113.2589 E; 24.5089 N	690m	Q. Fan	14797	
GDZMP	*E. tonkinensis*	China	Guangdong Province: Tongguzhang Mountain	116.6072 E; 24.3219 N	820m	C. Y. Huang; Y. R. Liu; F. Ye	HCY180604	SYS00237066
GDFXC	*E. tonkinensis*	China	Guangdong Province: Wudong Mountain, Chaozhou City	116.6571 E; 23.9630 N	894m	C. Y. Huang; Y. R. Liu; F. Ye	HCY180605	SYS00237014; SYS00237015
GDBX	*E. tonkinensis*	China	Guangdong Province: Baixi Nature Reserve, Heyuan City	115.2169 E; 23.7149 N	703m	C. Y. Huang; Y. R. Liu; F. Ye	HCY1725	
GXLJ	*E. tonkinensis*	China	Guangxi Province: Liujufan Village, Laibin City	110.0286 E; 23.9132 N	980m	Q. Fan	16641	SYS00237009; SYS00237010

GXGPS	*E. tonkinensis*	China	Guangxi Province: Guposhan Mountain, Hezhou City	110.5414 E; 24.6185 N	650m	Q. Fan	HCY1813	SYS00237058
HAIJFL	*E. tonkinensis*	China	Hainan Province: Jianfengling National Forest Park	108.8729 E; 18.7171 N	1300m	Q. Fan	14150	
HAIJX	*E. tonkinensis*	China	Hainan Province: Jiaxi Nature Reserve	109.1514 E; 18.8856 N	1247m	Q. Fan	HCY190802	
HAILMS	*E. tonkinensis*	China	Hainan Province: Limushan Mountain National Forest Park	109.7535 E; 19.1817 N	954m	Q. Fan	14136	
HAIDLS	*E. tonkinensis*	China	Hainan Province: Diaoluo Mountain National Forest Park	109.8829 E; 18.709 N	782m	Q. Fan	14115	
HK	*E. tonkinensis*	China	Hong Kong SAR: Wong Lung Hang	113.9577 E; 22.2606 N	518m	Q. Fan; C. Y. Huang; Q. Y. Yin;	17416	
HNMJX	*E. tonkinensis*	China	Hunan Province: Maojixian Nature Reserve	113.7314 E; 26.0797 N	804m	W. Y. Zhao	HCY1604	
HNQYF	*E. tonkinensis*	China	Hunan Province: Qiyunfeng National Forest Park	113.9389 E; 25.7743 N	671m	W. Y. Zhao	LXP-13-25645	
HNJQS	*E. tonkinensis*	China	Hunan Province: TaoyuandongTaoyuandong Nature Reserve	114.0764 E; 26.564 N	761m	W. Y. Zhao	HCY1614	
JXWZF	*E. tonkinensis*	China	Jiangxi Province: Wuzhifeng National Forest Park	114.0355 E; 26.9119 N	1081m	W. Y. Zhao	HCY1714	
JXJPS	*E. tonkinensis*	China	Jiangxi Province: Jinpenshan National Forest Park	115.2316 E; 25.2197 N	571m	W. Y. Zhao	HCY1642	
GXYRT	*E. populnea*	China	Guangxi Province: Yanrong Town, Baise City	105.9543 E; 23.1289 N	1102m	W. Y. Zhao	ZWY-634	SYS00237001; SYS00237002;
GXWDX	*E. populnea*	China	Guangxi Province: Baxiongshan Mountain, Baise City	105.7819 E; 23.2860 N	1280m	Q. Fan	16596	
GXBXS	*E. populnea*	China	Guangxi Province: Sanfang Town, Liuzhou City	108.8793 E; 25.2624 N	678m	W. Y. Zhao	ZWY-623	
GZLB	*E. populnea*	China	Guizhou Province: Beside Y011 National Highway, Lipo Country	108.0893 E; 25.2706 N	486m	C. Y. Huang; L. Y. Wang	HCY1601	SYS00237053; SYS002370534
GZGHX	*E. populnea*	China	Guizhou Province: Guanghui Village, Congjiang Country	108.347 E; 25.6344 N	676m	W. Y. Zhao	ZWY-446	SYS00237059

XZ01	*E. populnea*	China	Xizang (Tibet): Motuo County	95.1702 E; 29.2154 N	1769m	Q. Fan	14361	
XZ02	*E. populnea*	China	Xizang (Tibet): Motuo County	95.4467 E; 29.4903 N	865m	Q. Fan	14312	
YNQTZ	*E. populnea*	China	Yunan Province: Qiaotou Town, Honghe Prefecture	101.1231 E; 22.8547 N	960m	Q. Fan	15331	
YNXLS	*E. populnea*	China	Yunan Province: Xilongshan Mountain, Honghe Prefecture	102.8028 E; 22.6764 N	2190m	Q. Fan	16753	
YNDWS	*E. populnea*	China	Yunan Province: Daweishan Mountain National Nature Reserve	103.6993 E; 22.9195 N	1970m	Q. Fan	15342	
YNMBZ	*E. populnea*	China	Yunan Province: Laojunshan Mountain, Wenshan Prefecture	103.9659 E; 23.3028 N	1943m	W. Y. Zhao	ZWY-785	
YNJTC	*E. populnea*	China	Yunan Province: Jiutou Village, Wenshan Prefecture	104.1915 E; 23.3814 N	1423m	Q. Fan	16001	
YNQLD	*E. populnea*	China	Yunnan Province: Qinlangdang (Maku Villey), Nujiang Prefecture	98.2878 E; 27.6822 N	1660m	W. Y. Zhao	ZWY-866	
YNGLG	*E. populnea*	China	Yunnan Province: Gaoligong Mountain, Nujiang Prefecture	98.5692 E; 27.7515 N	1936m	W. Y. Zhao	ZWY-877	SYS00237035
YNYFS	*E. populnea*	China	Yunnan Province: Yunfengshan Mountain, Tengchong City	98.4133 E; 25.3790 N	2086m	Q. Fan	WHW618	
YNPMZ	*E. populnea*	China	Yunnan Province: Pianma Town, Lushui City	98.6410 E; 26.0166 N	2100m	Q. Fan	16650	SYS00237007
YNHQZ	*E. populnea*	China	Yunnan Province: Houqiao Town, Tengchong City	98.1398 E; 25.5158 N	2880m	W. Y. Zhao	ZWY-819	SYS00236999; SYS00237000;
YNBLF	*E. populnea*	China	Yunnan Province: Bailaofeng Moutain, Baoshan City	98.7697 E; 25.1325 N	2500m	Q. Fan	16660	
NP	*E. populnea*	Nepal	Kosi Zone: Border with Sunsari and Morang	87.5575 E; 26.8758 N	1622m	Q. Fan；K.K. Meng	17060	
VNTPO	*E. populnea*	Vitnam	Cao Bằng Province: Phia Den National Park	105.8759 E; 22.5882 N	1240m	Q. Fan；Q. Y. Yin	17213	SYS00237012
VNNCS	*E. populnea*	Vitnam	Lào Cai Province: Nhiu Co San Village	103.6255 E; 22.6269 N	1833m	Q. Fan；Q. Y. Yin	17260	

VNSSH	*E. populnea*	Vitnam	Lào Cai Province: San Sả Hồ	103.7779 E; 22.3589 N	1814m	Q. Fan; Q. Y. Yin	16452	SYS00237005; SYS00237006
VNSL	*E. populnea*	Vitnam	Son La Province: Cepia Nature Reserve	103.5869 E; 21.3335 N	1484m	Q. Fan; Q. Y. Yin	16492	SYS00237013
IN	*E.tricuspis*	Indonesia	Sumatra: Mt. Kenınci	101.2607 E; 1.7341 S	2054m	Q. Fan; S. Shi	15756	SYS00236998
MATR	*E.tricuspis*	Malaysia	Perak State: ameron Highland Tanah Rata	101.3785 E; 4.4731 N	1436m	Q. Fan; S. Shi	16803	
MAFH	*E.tricuspis*	Malaysia	Selangor State: Near to Fraser's Hill	101.7396 E; 3.6919 N	970m	Q. Fan; S. Shi	16800	SYS00236997
VNKB	*E.tricuspis*	Vitnam	Kon TumKon Plông District: Kon Buo	108.2702 E; 14.5944 N	1168m	Q. Fan; Q. Y. Yin	17338	
VNBNB	*E.tricuspis*	Vitnam	Lâm Đồng Province: Bidoup-Nui Ba National Park	108.6901 E; 12.1853 N	1290m	Q. Fan; Q. Y. Yin	17370	
VNBHH	*E.tricuspis*	Vitnam	Quảng Trị Province: Bắc Hướng Hóa Nature Reserve	106.5852 E; 16.8014 N	1008m	Q. Fan; Q. Y. Yin	17303	SYS00237011
VMBM	*E.tricuspis*	Vitnam	Thua Thien Hue Province: Vườn quốc gia Bạch Mã (Bạch Mã National Park)	107.8618 E; 16.1969 N	1389m	Q. Fan; Q. Y. Yin	17306	SYS00237003
GXSW	*E. longipetala*	China	Guangxi Province: Shiwandashan National Forest Park	107.8953 E; 21.8434 N	508m	W. Y. Zhao	ZWY-495	SYS00237045 SYS00237046; SYS00237047; SYS0023749; SYS00237050
GZLGS	*E. longipetala*	China	Guizhou Province: Leigongshan National Nature Reserve	108.3025 E; 26.3300 N	825m	W. Y. Zhao	ZWY-401	SYS00237038; SYS00237039; SYS00237041; SYS00237042; SYS00237041
HNQJD	*E. longipetala*	China	Hunan Province: Qianjiadong National Forest Park	111.3367 E; 25.4398 N	490m	W. Y. Zhao	HCY2114	SYS00237057
HNBMS	*E. longipetala*	China	Hunan Province: Bamianshan Mountain National Nature Reserve	113.7130 E; 25.9778 N	1136m	W. Y. Zhao	HCY1629	

Supplementary Table S7. Development of chloroplast primers

Step	Tm(℃)
1	Select an *Exbucklandia* chloroplast genome assemble from high-throughput and aligned it with a chloroplast genome of *Rhodoleia championii* downloaded from NCBI database (NCBI:NC_045276.1) by MAFFT
2	Put aligned sequences in DnaSP Version 6.0 to find highly variable regions. Using the 'Sliding Window' tool to calculate single nucleotide polymorphisms (Pi) for each region, Windows Length set to 1000 bp and Step size to 200 bp. Based on Pi values, finally seven regions of Exbucklandia chloroplast genome with the highest polymorphisms were selected: EXB5, EXB7, EXB10, EXB12, EXB17, EXB18, EXB24.
3	All seven chloroplast regions then were imputed into Primer3 (https://primer3.ut.ee/) to design primers with a target length of 800 bp.
4	A total of 21 pairs were designed, they then were amplificated on *Exbucklandia* individuals (from 3 species). Finally, four primer pairs with high specificity and versatility were selected.

Supplementary Table S8. Chloroplast primers used in this study

Locus	Regions	Primer sequence（5' to 3'）	Tm(℃)
EXB5_1	*trn*S-*psb*Z	F: CCAATGCTTGAAGTAGGAATC<br>R: GACCAACCATCAGGAGAAG	55
EXB5_3	*trn*G-*trnf*M-*rps*14	F: GTGTGGACATATTGCGTATC<br>R: AAGTTCCGTCGTTGAGTG	52
EXB7_3	*trn*V	F: ATTGCGTTAGGTTGGACTT<br>TTAAGCAGAGCGATAGAGAC	53
EXB17_2	*rpl*32	F: GCGAATCCTACGAATCCTAT<br>R: CACACCATAAGAGCGAAGA	52

Supplementary Table S9. Characteristics of 21 SSR loci used in this research.

Locus	Motif	Primer sequence 5'-3'	Length (bp)	Tm(°C)	Putative function
EX301	(ATA)17	F:ATCACATGCGATGATAGCCC<br>R:CAAGCATGCCTAGAATCTTTGA	238-289	60	-
EX302	(AAG)16	F:GCGCAAAAATATGCACATACA<br>R:GAAATAACCCAAGCCCCTTC	130-181	60	-
EX327	(AGA)11	F:GGCTGCATTTGGTTGCTACT<br>R:GAGTCGGCGGAATTAAGTTG	233-266	60	*Exbucklandia populnea* 26S ribosomal RNA gene, complete sequence
EX336	(TTC)10	F:CAAGCAAGGTCTTCTCTTGGA<br>R:TTTTAGATTCCCAAAAATCGC	163-196	59	-
EX337	(TTC)10	F:GGTTCTGGGTCAGTCTCCAA<br>R:CCCTGCTACAATTCCTCCAA	198-225	60	-
EX346	(TCT)10	F:CAGAAGAAGGTGGTCAAGCC<br>R:AATCCATTCGCATCCATTGT	187-220	60	-
EX353	(CTT)9	F:TCAAGTCCTTTTGGCGAGTT<br>R:GCCGAAGAAGAAGAACGATG	249-279	60	-
EX357	(TCT)9	F:CCAAACGCTACAAGCAACTG<br>R:TGAATTCTTGACCGGAAACTG	234-282	60	-
EX358	(ATG)9	F:GGTCTGTTTCATTGTCCCGT<br>R:CCATCATTTTTGAATAGGGCA	237-273	60	PREDICTED: Prunus persica partner of Y14 and mago (LOC18790315), mRNA
EX359	(AAT)9	F:CTCAGCCCGAGTTAAGTTGA<br>R:GCTGGCAACGATGTTAGAGG	234-279	60	-
EX365	(CTT)10	F:AGCAGAAGGAGCAACAAGGA<br>R:GATCTCGACCGATCGTCACT	224-269	60	-
EX378	(AAT)9	F:CATTGGCCTCTCTAGGCATC<br>R:ACAGTAGCATGGCTGCATCA	197-233	60	-
EX379	(AAT)9	F:AAAATAATGGTAATTTGTGGTTTTGA<br>R:AAAGGTGTTGGATATTTCTTTACATTT	185-218	59	-
EX403	(TATG)8	F:AAAGAAAATGGGATGGAACG<br>R:TCCTTCCTCAGGTGGAACAC	250-286	59	-
EX406	(ATGG)7	F:TCCGCCCTAATCTTTCTTCA<br>R:AGGGACTATGGACCATTCACA	173-201	60	-
EX410	(TATG)7	F:AACTTCCGGCATTCAATGAT<br>R:TGGGGTACTGGGATCAATTC	138-158	60	-
EX506	(TTCTT)7	F:TGCTTAAAATCACCAATTCAGATG<br>R:ATGCCCTTCTTGGCATTTTA	146-181	60	PREDICTED: *Vitis vinifera* UDP-galactose transporter 2 (LOC100265002), mRNA
EX608	(CCATCA)6	F:GTTCTTCTTTCCTCCAGCCC<br>R:CGGAGGCAATGGAAAAATTA	221-287	60	-
EX609	(GAGAAG)6	F:GCCATCCTCTTAAATTTTCCC<br>R:AAGACACCCAAACTTGCTGC	227-269	60	PREDICTED: *Populus trichocarpa* pollen receptor-like kinase 4 (LOC7465580) , mRNA
EX610	(GGGTTA)6	F:CAGGACCCACCAACTGAAG<br>R:GGTTTGGTGGGGGTTAAAGT	225-279	60	-
EX6511	(ATACCT)5	F:GAAATTGCTGACAGGAAGGC<br>R:TGAATTCTGACGGTCACTGC	237-285	60	-
